# Restoration of histone acetylation ameliorates disease and metabolic abnormalities in a FUS mouse model

**DOI:** 10.1186/s40478-019-0750-2

**Published:** 2019-07-05

**Authors:** Elisabeth Rossaert, Eveliina Pollari, Tom Jaspers, Lawrence Van Helleputte, Matthew Jarpe, Philip Van Damme, Katrien De Bock, Matthieu Moisse, Ludo Van Den Bosch

**Affiliations:** 1 Department of Neurosciences, Experimental Neurology, and Leuven Brain Institute (LBI), KU Leuven – University of Leuven, Herestraat 49, 3000 Leuven, Belgium; 2 VIB, Center for Brain & Disease Research, Laboratory of Neurobiology, Leuven, Belgium; 3 Regenacy Pharmaceuticals Inc., Waltham, MA USA; 4 Department of Neurology, University Hospitals Leuven, Leuven, Belgium; 5 Laboratory of Exercise and Health, Department of Health Sciences and Technology, ETH Zurich, Zurich, Switzerland

**Keywords:** Amyotrophic lateral sclerosis, Neurodegeneration, FUS, Histone deacetylases, HDAC inhibitors, Epigenetics, Metabolism

## Abstract

Dysregulation of epigenetic mechanisms is emerging as a central event in neurodegenerative disorders, including amyotrophic lateral sclerosis (ALS). In many models of neurodegeneration, global histone acetylation is decreased in the affected neuronal tissues. Histone acetylation is controlled by the antagonistic actions of two protein families –the histone acetyltransferases (HATs) and the histone deacetylases (HDACs). Drugs inhibiting HDAC activity are already used in the clinic as anti-cancer agents. The aim of this study was to explore the therapeutic potential of HDAC inhibition in the context of ALS. We discovered that transgenic mice overexpressing wild-type *FUS* (“Tg *FUS*+/+”), which recapitulate many aspects of human ALS, showed reduced global histone acetylation and alterations in metabolic gene expression, resulting in a dysregulated metabolic homeostasis. Chronic treatment of Tg *FUS*+/+ mice with ACY-738, a potent HDAC inhibitor that can cross the blood-brain barrier, ameliorated the motor phenotype and substantially extended the life span of the Tg *FUS*+/+ mice. At the molecular level, ACY-738 restored global histone acetylation and metabolic gene expression, thereby re-establishing metabolite levels in the spinal cord. Taken together, our findings link epigenetic alterations to metabolic dysregulation in ALS pathology, and highlight ACY-738 as a potential therapeutic strategy to treat this devastating disease.

**Electronic supplementary material:**

The online version of this article (10.1186/s40478-019-0750-2) contains supplementary material, which is available to authorized users.

## Introduction

Amyotrophic lateral sclerosis (ALS) is a fatal neurodegenerative disorder caused by the selective loss of motor neurons in the motor cortex, brainstem and spinal cord, leading to the paralysis of voluntary muscles [51]. Motor symptoms usually appear in midlife and ultimately escalate to death, usually within 2 to 5 years after symptom onset [51]. Approximately 90% of ALS cases are sporadic, while the remaining 10% are inherited [51]. The most prevalent genetic causes of ALS are mutations in the genes encoding superoxide dismutase 1 (SOD1), TAR DNA-binding protein 43 (TDP-43) and fused in sarcoma (FUS), as well as hexanucleotide repeats in the ‘*Chromosome 9 open reading frame 72*’ (*C9orf72*) gene [2]. Despite the identification of multiple genetic factors, the pathogenic mechanisms underlying the selective degeneration of motor neurons remain incompletely understood. At present, effective therapies are lacking for ALS patients [1, 9, 59]. As a consequence, there is a strong need for more effective therapies for ALS.

Mouse models manifesting the core symptoms of the disease, including a motor phenotype and motor neuron degeneration, remain crucial for the preclinical investigation of pathological mechanisms and novel therapeutic options. As all therapeutic strategies effective in the commonly used mutant SOD1 model subsequently failed in the clinic, other in vivo models to study ALS are urgently needed. One example is the PrP-hFUS-WT3 mouse model, which shows a strong ALS-like phenotype with severe motor neuron degeneration and a short lifespan, unlike many other FUS models [40, 62]. FUS is a DNA/RNA binding protein linked to ALS as well as to frontotemporal dementia (FTD), another neurodegenerative disorder belonging to the same disease spectrum as ALS [21, 69]. Mutations in the *FUS* gene account for approximately 4% of familial ALS cases and lead to neuronal and glial cytoplasmic mislocalization of the FUS protein [32, 70]. The majority of the identified mutations are found in the glycine-rich region and in the nuclear localization signal [21]. However, mutations in the 3′ untranslated region causing a strong increase in FUS expression have also been identified in ALS patients [19, 57]. Moreover, FUS aggregates are found in the absence of mutations in the *FUS* gene in a subset of FTD cases [16, 42]. These findings strongly indicate that not only mutant, but also wild-type FUS can be detrimental to neurons. However, it remains unclear which pathogenic mechanisms contribute to the observed neurodegeneration [40].

Recently, aberrant homeostasis of epigenetic marks was detected in the nervous system of several models of neurodegenerative disorders, including ALS [31, 34, 53, 58, 68]. One of these deregulated marks is acetylation of lysine residues of histones. Acetylation of histones regulates high-order chromatin folding and thereby the accessibility of transcription factors to their target genes [22]. Histone acetylation levels are regulated by the counteracting activities of two protein families –the histone acetyltransferases (HATs) and the histone deacetylases (HDACs) [22]. Accumulating evidence indicates that decreased histone acetylation is a negative determinant for neuronal survival [34, 53, 58, 68]. A major indication is that global histone hypoacetylation in the nervous system is a feature of multiple neurodegenerative models [7, 31, 58]. This imbalance tremendously affects the global transcription profile [53, 54, 58, 68].

HDACs are druggable targets and compounds that inhibit this class of enzymes are used to treat several forms of cancer [24]. The therapeutic potential of HDAC inhibitors was already studied in a wide range of neurodegenerative disorders, such as Alzheimer’s disease, Huntington’s disease, spinal muscular atrophy and ALS [5, 6, 37, 52, 54, 56, 64, 67, 76].

Many HDAC inhibitors were developed showing distinctive selectivity toward the 11 zinc-dependent HDACs identified in mammals. Genetic and pharmacological studies suggested that especially the class I HDACs (HDAC1, 2, 3 and 8) as well as the class IIb HDAC6 have the largest potential as treatment strategies for neurodegenerative diseases, including ALS [18, 34, 75]. Class I HDACs reside in the nucleus and their major targets are the histones. HDAC6 has distinct functions in the cytoplasm.

The therapeutic potential of pan-HDAC inhibitors has been investigated in SOD1 mice, showing modest effects. Principal determinants responsible for these limited outcomes could be the low HDAC inhibitory potency, poor blood-brain-barrier (BBB) permeability, lack of isoform selectivity or toxicity of the compounds used [54, 56, 64, 76]. These could also explain the negative results of phase II clinical trials with valproate and phenylbutyrate [13, 47]. Therefore, research on HDAC inhibition as a therapeutic strategy for ALS deserves further attention. Moreover, new HDAC inhibitors with improved characteristics were recently developed.

The aim of this study was to test the efficacy of the HDAC inhibitor ACY-738 in a transgenic *FUS* mouse model of ALS and to investigate the contribution of different types of HDACs to the disease phenotype. ACY-738 displays a selectivity profile towards class I HDACs and HDAC6 [8, 30, 41]. We used ACY-738 as it is unique amongst the HDAC inhibitors, having a high potency, tolerability, and capacity to penetrate the BBB [30, 37, 41]. ACY-738 treatment restored histone acetylation and slowed down disease progression of the Tg *FUS*+/+ mice. This treatment also corrected metabolic pathways in the spinal cord, which were already dysregulated at presymptomatic stage. Overall, our data suggest that global histone hypoacetylation is associated with metabolic dysregulation in FUS-mediated ALS pathology, and that histone deacetylases are potential therapeutic targets to delay disease progression by re-establishing metabolic homeostasis.

## Material and methods

### Animals

Tg *FUS+/−* breeding mice (stock no. 017916) were purchased from The Jackson Laboratory (Maine, USA). From the age of 28 to 30 days, crushed Teklad LM-485 (7912) sterilizable rodent chow (Envigo, Cambridgeshire, UK) containing 100 mg/kg ACY-738 (Regenacy Pharmaceuticals Inc., Waltham, USA) and mixed with water was provided *ad libidum*.

For genotyping, DNA was isolated from ear biopsies, and digested overnight in lysis buffer with 20 mg/ml proteinase K (Roche, Basel, Switzerland) at 55 °C. Genotyping of the mice was done using qPCR with a FAM-labelled probe recognizing the human *FUS* transgene (forward and reverse primers 5′-CAGCAAAGCTATGGACAGC-3′ and 5′-GTCTTGATTGCCATAACCGC-3′ and Taqman probe 5′-AGCAGAACCAGTACAACAGCAGCA-3′). *β-actin* was used as a housekeeping gene (forward and reverse primers 5′-CCCTACAGTGCTGTGGGTTT-3′ and 5′-GACATGCAAGGAGTGCAAGA-3′).

All mice were housed according to the guidelines of the KU Leuven. Mice were randomly selected for drug treatments and littermates were used as controls. Sample size and intermediate endpoints were based on the original study and a pilot experiment [40].

### ACY-738 bioavailability

The concentration of ACY-738 in plasma and brain samples was determined by Liquid Chromatography/tandem Mass Spectrometry (LC-MS/MS). Plasma and brain were collected from mice killed after 5 days of ACY-738 therapy. ACY-738 was extracted from the samples by protein precipitation using 50:50 acetonitrile:methanol and analyzed using LC/MS/MS. The concentrations were then calculated using a standard curve.

### Electrophysiology

Nerve conduction measurements were performed as described previously [14]. Briefly, mice were anaesthetized under a 2.2 l/min oxygen flow containing 3% isoflurane and placed on a heating pad (Physitemp Instruments Inc., Clifton, USA) to maintain the body temperature. Motor nerve conduction studies were performed using sub-dermal platinum coated 0.4 mm needle electrodes (Technomed Europe, Maastricht, the Netherlands) for stimulating and recording and a UltraPro S100 monitoring set-up (Natus Medical Incorporated, Pleasanton, USA). Compound muscle action potentials (CMAPs) were determined by measuring the electrical response of the gastrocnemius muscle after stimulation of the sciatic notch. The stimulation intensity was gradually increased until the supramaximal CMAP amplitude was obtained. The amplitude and latency of the signal were quantified. The average of 3 measurements was determined.

### Behavioral testing

Muscle force of the mice was measured using a Grip Strength Meter (Columbus Instruments, Columbus, USA) with a triangular bar as a probe to measure the grip strength in the forepaws. The average of five trials per animal was determined. To assess both fore- and hindlimb muscular strength, the hanging wire test was performed. Mice were placed on a wire cage lid. The lid was inverted and the latency to fall was recorded to a maximum of 60 s. The average of three trials per animal was determined. We evaluated motor performances and weight two times a week starting at 28 days of age. Disease onset was considered as the time point at which the mouse showed the highest value for forelimb grip strength. Survival was determined by the loss of righting reflex within 5 s after laying the mouse on its side. This time point was considered as disease end-stage, whereupon the mouse was sacrificed by intraperitonial injection with 200 mg/kg sodium pentobarbital (Dolethal, Vetoquinol) and used for histopathology.

### Histopathology

Mice were anaesthetized by intraperitonial injection with 200 mg/kg sodium pentobarbital (Dolethal, Vetoquinol) and transcardially perfused with PBS.

For spinal cord histology, the lumbar part of the spinal cord was harvested and post-fixed with 4% paraformaldehyde (PFA) overnight (4 °C) and dehydrated for 48 h in 30% sucrose (4 °C). To visualize the motor neurons in the ventral horn, cryosections (20 μm) were stained with a 2X thionin solution. Briefly, slides were washed twice with water for 15 s, stained with 2X thionin for 45 s, again washed twice with water and dehydrated in a three-step ethanol series of increasing concentrations (70, 90 and 100%) for 30 s each. Sections were cleared with Histoclear solution for 1 min and mounted with PerTex mounting medium (Histolab, Göteborg, Germany). Images were acquired with a Zeiss Imager M1 microscope (Carl Zeiss, Oberkochen, Germany) using a 10x objective. The area of neuron cell bodies in the ventral horn were analyzed using the AxioVision program and neurons larger than 400 μm^2^ were considered as motor neurons. To investigate FUS localization in the spinal cord, colocalization of FUS with neurons was assessed. Sections (20 μm) were blocked for 1 h with 10% Normal Donkey Serum (Sigma, St. Louis, USA) in PBS-T (0.1%) and incubated overnight (4 °C) with antibodies for FUS (Proteintech 11,570-A-1P, 1:100) and NeuN (Millipore, MAB377; 1:500). The sections were subsequently washed and incubated with appropriate secondary antibodies (ThermoFisher Scientific, 1:1000). Slides were mounted with DAPI-containing ProLong® Gold antifade reagent (Invitrogen - Life Technologies, Carlsbad, USA). To investigate astrocytosis and microgliosis, a similar protocol using antibodies against GFAP (Sigma, G3893; 1:200), CD11b (Serotex MCA74G; 1:200) was used. Images were acquired using the Leica SP8x confocal microscope.

For muscle histology, the legs were tied with a strip during perfusion. The gastrocnemius muscles were dissected and snap-frozen in isopentane cooled by immersion in liquid nitrogen. To visualize neuromuscular junctions, longitudinal cryosections (20 μm) were fixed with 4% PFA for 10 min and then washed with PBS and PBS-T (0.1% Triton X-100). Subsequently, sections were blocked for 1 h with 10% Normal Donkey Serum (Sigma) in PBS-T. To visualize the nerve axons, the sections were incubated overnight (4 °C) with Neurofilament-L conjugated to Alexa-488 antibody (Cell Signaling Technologies; 1:500), Synaptophysin antibody (Cell Signaling Technologies; 1:500) and Synaptic vesicle 2 (DSHB; 1:100). The sections were then washed and incubated with appropriate secondary antibodies (ThermoFisher Scientific, 1:1000) and α-bungarotoxin conjugated to Alexa-555 (Invitrogen; 1:1000) for 1 h at room temperature (RT). Slides were mounted with ProLong® Gold antifade reagent (Invitrogen - Life Technologies). At least 50 neuromuscular junctions were analyzed for innervation, as determined by the co-localization of the Neurofilament-L/Synaptophysin/Synaptic vesicle 2 and α-bungarotoxin labeling, on a Zeiss Axio Imager M1 microscope (Carl Zeiss) using a 10x objective. To visualize muscle fibers, transversal cryosections (20 μm) were blocked for 1 h with 1% BSA (Serva, Heidelberg, Germany) in PBS, after which they were incubated with WGA conjugated to Alexa-488 antibody (1/100 Invitrogen W11261) in 0.5% BSA in PBS for 1 h at room temperature. After washing three times with PBS, slides were mounted with ProLong® Gold antifade reagent (Invitrogen - Life Technologies).

### Western blot analysis

For blotting of acetylated α-tubulin or FUS, tissue samples were lysed in RIPA buffer (50 mM Tris-HCl (pH 7.5), 150 mM NaCl, 1% NP-40, 0.5% Na-deoxycholic acid, 0.5% SDS), supplemented with Complete EDTA-free protease inhibitor cocktail (Roche, Basel, Switzerland). Tissues were homogenized using Lysing Matrix D beads (MP Biomedicals, Solon, USA) and a MagNa Lyser oscillator (Roche) at 6500 rpm for 30 s thrice with 1 min interval on ice. The samples were subsequently centrifuged at 14,000 rpm for 20 min, after which the supernatant was transferred to a pre-chilled tube.

For blotting of acetylated histones and for the subcellular fractionation experiment, tissue samples were extracted using the NE-PER Nuclear and Cytoplasmic Extraction kit (Pierce Biotechnology, Rockford, USA) following the Manufacturer’s instructions. Briefly, tissues were cut in small pieces, washed with PBS and homogenized with a pellet pestle motor in CER I buffer. Samples were vortexed thoroughly for 15 s and left on ice for 10 min. CER II buffer was added, after which the mixture was vortexed and centrifuged at 16,000 g for 5 min. The nuclear pellet was washed with CERI/II buffer, after which it was resuspended in NER buffer. Samples were left on ice for 40 min, vortexing thoroughly for 15 s every 10 min. All samples were sonicated quickly, centrifuged at 16,000 g for 10 min and the supernatant (nuclear extract) was transferred to a pre-chilled tube. For histone blotting, only the nuclear extract was used.

Protein concentrations were measured with the micro BCA kit (Pierce Biotechnology). Reducing sample buffer (ThermoFisher Scientific) was added to samples containing equal amounts of protein (2.5 μg for α-tubulin; 30 μg for FUS; 15 μg for histones) and heated for 10 min at 95 °C before separation on a SDS–polyacrylamide electrophoresis gel (12%, 90 V, 50 min for α-tubulin; 3 h for FUS). Proteins were transferred to a polyvinylidene difluoride (PVDF) membrane (Millipore, Massachusetts, USA) by a semi-dry transfer apparatus (Bio-Rad, Hercules, USA) (180 mA, 1 h 45 min). The membranes were blocked with 5% milk in TBS-T (10 mM Tris-HCl (pH 7.5), 150 mM NaCl and 1% Tween-20) for 1 h at RT. Immunoblots were then incubated with primary antibodies against FUS (rabbit, 1/1000, Bethyl A300–302), acetylated α-tubulin (mouse, 1/5000, Sigma T6793), calnexin (rabbit, 1/2000, Enzo Life Technologies ADI-SPA-860-F), acetyl histone 3 K9/14 (rabbit, 1/500, Cell Signaling 9677), histone 4 (rabbit, 1/500, Abcam ab10158) in TBS-T for 1 to 2 h at RT. The membranes were incubated with the appropriate secondary antibody conjugated with horseradish peroxidase (HRP) (1/5000; Agilent Technologies (Dako)) for 1 h at RT. Protein bands were visualized using enhanced chemiluminescence (ECL substrate, ThermoFisher Scientific) and an ImageQuant LAS 4000 Biomolecular Imager (GE Healthcare, Illinois, USA). For histone blotting, a mild reblotting buffer (Millipore) was applied to strip the blots. Band intensities were quantified using ImageQuant™ TL version 7.0 software (GE Healthcare) and normalized to the appropriate control (calnexin or histone 4).

### In situ nuclear HDAC activity

For the determination of in situ nuclear HDAC activity, nuclei of tissue samples were extracted using the Epiquick Nuclear Extraction Kit (Epigentek, Farmingdale, USA), after which HDAC activity was determined using the Epigenase HDAC Activity/Inhibition Direct Assay Kit (Colorimetric) (Epigentek). In brief, tissues were homogenized in NE1 buffer using a motor-driven pestle. After 15 min incubation on ice, the samples were centrifuged for 10 min at 12,000 rpm, after which the supernatant was removed. Two volumes of NE2 containing PIC were added to the pellet, after which the samples were incubated on ice for 15 min. Samples were vortexed, sonicated and centrifuged for 10 min at 14,000 rpm. The supernatant was collected and the protein concentration was measured with the micro BCA kit (Pierce Biotechnology). The HDAC activity in the nuclear extracts was then determined using the HDAC activity assay kit, by adding equal amounts of protein of the nuclear extracts in the wells of a microplate, together with HO1 and HO2 in a final volume of 50 μl. The plate was then incubated at 37 °C for 90 min, after which the reactions were removed from the wells. After thorough washing, primary antibodies were added to the wells for 60 min at RT, after which the secondary antibodies were incubated for 30 min at RT. After thorough washing, DS buffer was added for 5 min, after which SS buffer was added to stop the enzymatic reaction. Signals were detected by absorbance at 450 nm. The amount of deacetylated histone product was extrapolated from a standard curve, after which the HDAC activity in OD/min/mg was calculated.

### Transcriptional analysis

Mice were euthanized with CO_2_ followed by cervical dislocation. Spinal cord was rapidly dissected and snap-frozen in liquid nitrogen. RNA was extracted using a combined protocol of TRIzol:chloroform and the RNeasy mini kit (Qiagen) and further processed according to the manufacturer’s protocol. For RNA sequencing, samples were sent to the Nucleomics Core (VIB, Leuven, Belgium) and analyzed for RNA integrity (≥ 8) by running on a Bioanalyzer (Agilent) before Trueseq total stranded RNA library preparation and sequencing on a Illumina NextSeq 500 system (Illumina, San Diego, USA). The libraries were sequenced using a high output paired end with 75 bp reads and ~ 50 million reads per sample. Reads were mapped using STAR aligner [20] on the latest mouse genome build (mm10). Reads were then counted using Salmon to estimate transcript and gene expression of every sample [80]. Differential expression of coding genes and transcripts was performed with edgeR [38]. Gene and transcripts with a FDR-adjusted *P*-value smaller than 0.05 and with an altered expression of 30% were deemed significantly differentially expressed. Differentially expressed genes were used as input for Panther gene ontology enrichment analysis to identify pathways associated with the differentially expressed genes [3, 39, 66]. Multiple testing correction was performed using FDR Benjamini-Hochberg correction. The most significant and promising genes were validated with qRT-PCR. For qRT-PCR analysis, first-strand cDNA was synthesized using SuperScript III (Invitrogen). PCR reactions were performed using SybrGreen reagents (ThermoFisher Scientific) with primers specific for the genes-of-interest (Additional file 1: Table S1) [63]. Expression levels were normalized to two reference genes: AP3d1 (forward, 5′-CAAGGGCAGTATCGACCGC-3′; reverse, 5′-GATCTCGTCAATGCACTGGGA-3′) and Mon2 (forward, 5′-CTACAGTCCGACAGGTCGTGA-3′; reverse, 5′-CGGCACTGGAGGTTCTATATCTC-3′). Analysis was performed using qBase+ (v.3.0, Biogazelle, Zwijnaarde, Belgium).

### Proteomics analysis

Mice were anaesthetized by intraperitonial injection with 200 mg/kg sodium pentobarbital (Dolethal, Vetoquinol, UK) and transcardially perfused with PBS (Sigma). Spinal cord was rapidly dissected and snap-frozen in liquid nitrogen. Samples were sent to the Proteomics Core Facility (VIB, Ghent, Belgium) for Mass Spectrometry analysis. Liquid Chromatography - tandem Mass Spectrometry (LC-MS/MS) runs of all samples were searched together using the MaxQuant algorithm (version 1.6.1.0) with mainly default search settings including a false discovery rate set at 1% on both the peptide and protein level. Spectra were searched against the human protein sequences in the Swiss-Prot database (database release version of January 2018 containing 20,234 human protein sequences) (http://www.uniprot.org). Proteins that were identified in all replicates of at least one condition were kept. For statistical analysis, the missing values were imputed using a mixed approach. Missing not at random (MNAR) values were imputed using quantile regression-based left-censored function (MLE-QRILC), while missing at random values (MAR) were imputed using maximum-likelihood estimation (MLE). MNAR was assumed when the protein was not identified in all replicates of at least one condition. To reveal proteins of which the expression level was significantly regulated between the different conditions, the test_diff function of the DEP R-package was performed to compare intensities of the proteins between the three different conditions (non-Tg, Tg *FUS*+/+ vehicle, Tg *FUS*+/+ ACY-738) [78].

### Metabolomics analysis

Mice were euthanized with CO_2_ followed by cervical dislocation. Spinal cord was rapidly dissected and snap-frozen in liquid nitrogen. Samples were sent to the Metabolomics core (VIB, Leuven, Belgium) to perform targeted metabolomic profiling by Liquid Chromatography - Mass Spectrometry (LC-MS). Separation of metabolites prior to MS measurement was performed using a Dionex UltiMate 3000 LC System (Thermo Scientific) in-line coupled to a Q Exactive Orbitrap mass spectrometer (Thermo Scientific). Practically, 10 μl of the extract was injected on a C18 column (Acquity UPLC®HSS T3 1.8 μm 2.1x100mm, Waters) using solvent A (H_2_O, 10 mM Tributyl-Amine, 15 mM acetic acid) and solvent B (100% Methanol). Chromatographic separation was achieved with a flowrate of 0.250 ml/min and the following gradient elution profile: 0 min, 0%B; 2 min, 0%B; 7 min, 37%B; 14 min, 41%B; 26 min, 100%B; 30 min, 100%B; 31 min, 0%B; 40 min, 0%B. The column was thermostatted at 40 °C throughout the analysis. The MS operated in full scan negative ion mode (m/z range: 70–1050 Th) using a spray voltage of 4.2 kV, capillary temperature of 320 °C, sheath gas at 50.0, auxiliary gas at 15.0. The AGC target was set at 3e6 and resolution at 140,000, with a maximum IT fill time of 512 ms. Data processing was performed using the Xcalibur Quan software (Thermo Scientific).

### Statistical analyses

Statistical analyses were performed using GraphPad Prism software version 8.0.0 (GraphPad software Inc). For Kaplan-Meier survival curves, the log-rank test was used to determine the statistical significance. Unpaired two-tailed Student’s *t*-test was used for the comparison of two means. If needed, the results were corrected for multiple testing using the Holm-Sidak method, with Q = 1. One-way and two-way ANOVA were used for multiple group analyses. Data were tested for equal variances using Bartlett’s test, Brown-Forsythe test, F-test. Kruskal-Wallis and Mann-Witney were used when no equal variances were obtained in the data sets. Data are presented as means ± SEM. Statistical significance was set at *P* < 0.05. *P < 0.05, **P < 0.01, ***P < 0.001 ****P < 0.0001.

## Results

### Progressive neurodegeneration in Tg FUS+/+ mice is associated with hypoacetylation of histones

**Fig. 1:**
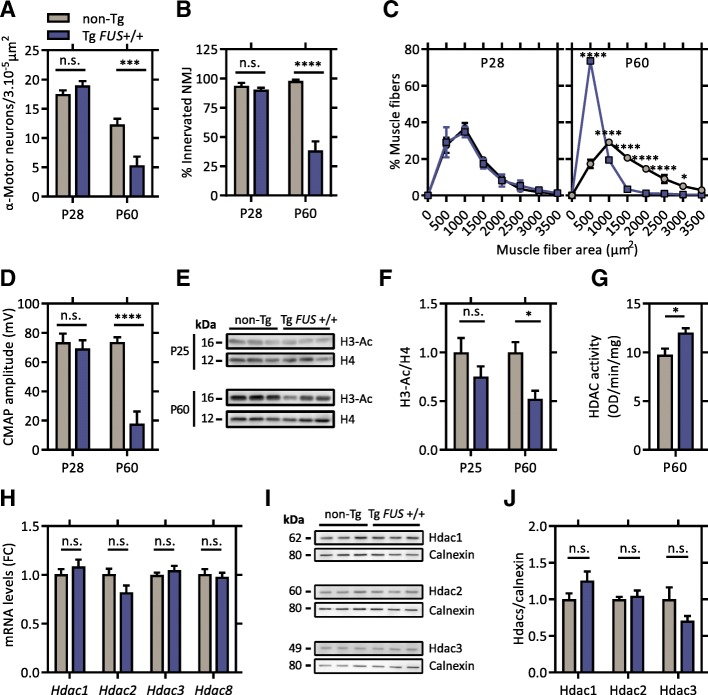
Neuronal histone hypoacetylation is associated with progressive neurodegeneration in Tg FUS+/+ mice. **a** Number of α-motor neurons in the spinal cord of P28 and P60 Tg *FUS*+/+ mice and non-Tg controls. *n* = 4–6, Student’s *t*-test. **b** Percentage of innervated neuromuscular junctions in the gastrocnemius muscle of P28 and P60 Tg *FUS*+/+ mice and non-Tg controls. *n* = 3–6, Student’s *t*-test. **c** Muscle fiber area distribution profile of the gastrocnemius muscle of P28 and P60 Tg *FUS*+/+ mice and non-Tg controls. *n* = 3, Two-way ANOVA with Tukey’s multiple comparisons test. **d** Compound muscle action potential amplitudes measured at the gastrocnemius muscle of P28 and P60 Tg *FUS*+/+ mice and non-Tg controls. *n* = 3–4, Student’s *t*-test. **e** Western blot of histone 3 acetylation levels in the spinal cord of P25 and P60 Tg *FUS*+/+ mice and non-Tg controls. Total histone 4 levels were used as reference for equal loading. **f** Quantification of the ratio of acetylated histone 3 to total histone 4 levels and normalization to non-Tg controls. *n* = 3, Student’s *t*-test. **g** In situ nuclear HDAC activity measurement on spinal cord homogenates of P60 Tg *FUS*+/+ mice and non-Tg controls. *n* = 3, Student’s *t*-test. **h** Quantitative PCR analysis of mRNA expression levels of individual class I HDACs in the spinal cord of P60 Tg *FUS*+/+ mice and non-Tg controls, with Ap3b1 and Mon2 as reference genes and normalization to non-Tg controls. Fold change compared to non-Tg controls (FC). *n* = 6, Student’s *t*-test with Holm-Sidak method to correct for multiple testing. **i** Western blot of Hdac1, Hdac2 and Hdac3 in the spinal cord of P60 Tg *FUS*+/+ mice and non-Tg controls. Calnexin levels were used as reference for equal loading. **j** Quantification of the ratio of Hdac1, Hdac2 and Hdac3 to calnexin and normalization to non-Tg controls. *P < 0.05, ***P < 0.001, ****P < 0.0001. Data are presented as means ± SEM

To investigate the gradual deterioration of motor units in the Tg *FUS*+/+ mice, we assessed several characteristic neuropathological features before symptom onset (P28) and at late-symptomatic age (P60). Prior to the appearance of motor symptoms, there was no difference in the number of large α-motor neurons in the ventral horn of the lumbar spinal cord between Tg *FUS*+/+ mice and non-Tg littermate controls (Fig. 1a). At late-symptomatic age, approximately 60% of the α-motor neurons were lost in Tg *FUS*+/+ mice compared to age-matched controls (Fig. 1a). Assessment of the innervation of the gastrocnemius muscle showed a similar progressive neuromuscular denervation in Tg *FUS*+/+ animals compared to controls (Fig. 1b). Analysis of the myofiber size distribution in the gastrocnemius muscle showed a comparable profile as controls at pre-symptomatic age (Fig. 1c). However, a significant increase in small myofibers compared to controls was observed in Tg *FUS*+/+ animals at end-stage, indicating severe muscle atrophy (Fig. 1c). As a functional read-out of neuromuscular innervation, we performed in vivo nerve conduction studies in the sciatic nerve by measuring compound muscle action potentials (CMAPs). In line with our histological results, comparable amplitudes were measured at the gastrocnemius muscle in Tg *FUS*+/+ mice and controls at pre-symptomatic age, but these were significantly reduced at end-stage, complying with gradual axonal loss (Fig. 1d).

Interestingly, we found that the global acetylation levels of histones (lysine 9/14) were significantly reduced in spinal cord and cortical tissue of late-symptomatic (P60), but not of pre-symptomatic Tg *FUS*+/+ mice (P28) (Fig. 1e, f). To investigate whether the decreased histone acetylation was due to excessive activation of HDACs, we assessed the in situ enzymatic activity of nuclear HDACs in spinal cord homogenates of P60 mice (Fig. 1g). This assay revealed a 30% increase in nuclear HDAC activity in the spinal cord of Tg *FUS*+/+ mice compared to controls (Fig. 1g). Quantitative PCR and immunoblot analysis indicated no obvious expression changes of any of the class I HDACs, implying a post-transcriptional dysregulation of the HDACs (Fig. 1h, i and j).

### HDAC inhibition mitigates the ALS disease phenotype in Tg FUS+/+ mice

**Fig. 2:**
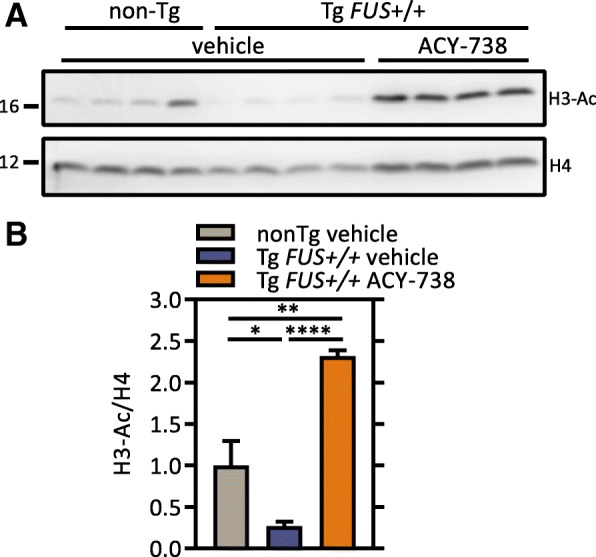
ACY-738 treatment restores histone acetylation. **a** Western blot of histone 3 acetylation levels in the spinal cord of P60 non-Tg controls, vehicle- and ACY-738-treated (100 mg/kg ACY-738 in chow) Tg *FUS*+/+ mice. Histone 4 levels were used as reference for equal loading. Hyperacetylation of histone 3 was used as a readout for class I HDAC inhibition. **b** Quantification of the ratio of acetylated histone 3 to total histone 4 levels and normalization to non-Tg controls. *n* = 4, One-way ANOVA with Tukey’s multiple comparisons test. *P < 0.05, **P < 0.01, ****P < 0.0001. Data are presented as means ± SEM

In order to test whether nuclear HDAC hyperactivity might play a role in the phenotype observed in the Tg *FUS*+/+ mice, we tested the therapeutic potential of HDAC inhibition. ACY-738 increases histone acetylation in a concentration dependent manner. While it has the highest potency to inhibit HDAC6, class I HDACs are also targeted at higher concentrations. In N2a cells, 3 μM ACY-738 administration significantly increases the acetylation of both α-tubulin (lysine 40) and histone 3 (lysine 9/14) (Additional file 2: Figure S1A, B). To determine the concentration at which ACY-738 inhibits class I HDACs in vivo*,* nuclear fractions of mouse spinal cord lysates were treated with increasing concentrations of ACY-738, followed by measurement of HDAC enzymatic activity. The half maximal inhibitory concentration (IC_50_) of ACY-738 for nuclear HDACs was 0.59 μM in the spinal cord (Additional file 2: Figure S1C). We then treated Tg *FUS*+/+ mice with ACY-738 for 5 consecutive days, after which we measured the drug concentration in blood and brain samples using Liquid Chromatography/Mass Spectrometry (Additional file 2: Figure S1D). The concentrations ranged from ~ 0.25 to ~ 0.50 μM (Additional file 2: Figure S1D). This is in the concentration window where ACY-738 can inhibit class I HDACs (Additional file 2: Figure S1C). Western blot analyses of spinal cord lysates of Tg *FUS*+/+ mice treated with ACY-738 indeed revealed a two- to threefold increase in histone H3 acetylation (lysine 9/14), validating inhibition of class I HDACs in the central nervous system (CNS) (Fig. 2a, b). Consistent with Fig. 1f, histone acetylation was decreased in untreated Tg *FUS*+/+ mice compared to non-Tg controls (Fig. 2a, b).

**Fig. 3:**
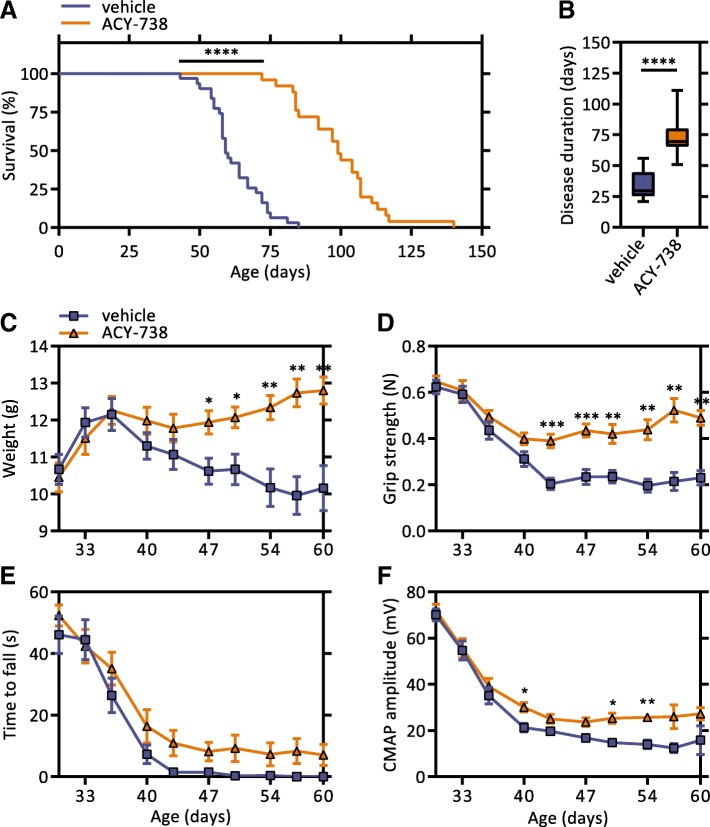
ACY-738 therapy ameliorates ALS disease phenotype in Tg *FUS*+/+ mice. **a** Kaplan-Meier survival analysis of vehicle- and ACY-738-treated (100 mg/kg ACY-738 in chow) Tg *FUS*+/+ mice. Median life span of vehicle-treated Tg *FUS*+/+ mice is 59 days, that of ACY-738-treated Tg *FUS*+/+ mice is 99 days. *n* = 23-25, of which 12 males and 11 females in the vehicle-treated group and 10 males and 15 females in the ACY-738-treated group, Log-rank test. **b** Disease duration of vehicle- and ACY-738-treated Tg *FUS*+/+ mice. *n* = 12–14, Two-tailed *t*-test. **c** Longitudinal follow-up of weight, **d** forelimb grip strength, **e** hanging wire performance, and **f** compound muscle action potential (CMAP) amplitudes of vehicle- and ACY-738-treated Tg *FUS*+/+ mice. *n* = 10–14, mice were followed up twice a week, Student’s *t*-test with Holm-Sidak method to correct for multiple testing. *P < 0.05, **P < 0.01, ***P < 0.001, ****P < 0.0001. Data are presented as means ± SEM

To investigate whether HDAC inhibition could improve the motor deficits of Tg *FUS*+/+ mice, ACY-738 was administered daily starting at symptom onset (P30) to mimic a clinically relevant time point. We monitored weight and motor function of ACY-738-treated and non-treated littermate Tg *FUS*+/+ mice twice a week during the disease course, as well as the survival. Strikingly, Kaplan-Meier survival analysis revealed that ACY-738-treatment led to a substantial 40 days increase in lifespan of Tg *FUS*+/+ mice, accounting for a 68% prolongation of survival (Fig. 3a). This was also reflected in the disease duration (defined as the time between disease onset and end-stage), which increased from a median of 29.5 days in non-treated to 73.5 days in ACY-738-treated Tg *FUS*+/+ mice (Fig. 3b). This beneficial effect on survival was seen both in males and females, with a larger effect in males (Additional file 3: Figure S2A, B). Treated mice also exhibited reduced weight loss compared to non-treated mice (Fig. 3c). Moreover, mice receiving ACY-738 showed improved motor performance, as evidenced by a significant increase in forelimb grip strength (Fig. 3d). Also a trend towards increased performance in the hanging wire test was detected (Fig. 3e). ACY-738 treatment likewise mitigated the reduction in CMAP amplitudes (Fig. 3f). Long-term follow up of ACY-738-treated mice revealed that end-stage treated mice acquired a comparable decrease in weight and forelimb grip strength as end-stage non-treated Tg *FUS*+/+ controls (Additional file 3: Figure S2C, D). To control for the compound treatment, we treated Tg *FUS*+/+ mice with ACY-1090, an inactive form of ACY-738. This drug has a very similar structure to ACY-738 but it is incapable of inhibiting its target HDACs as it has a modified zinc-binding group (Additional file 4: Figure S3A). Treatment with ACY-1090 did not demonstrate an effect on survival or on the motor performance of Tg *FUS*+/+ mice (Additional file 4: Figure S3B-E). All together, these findings show that HDAC inhibition by ACY-738 treatment ameliorated the disease phenotype and significantly extended the lifespan of the Tg *FUS*+/+ mice.

**Fig. 4:**
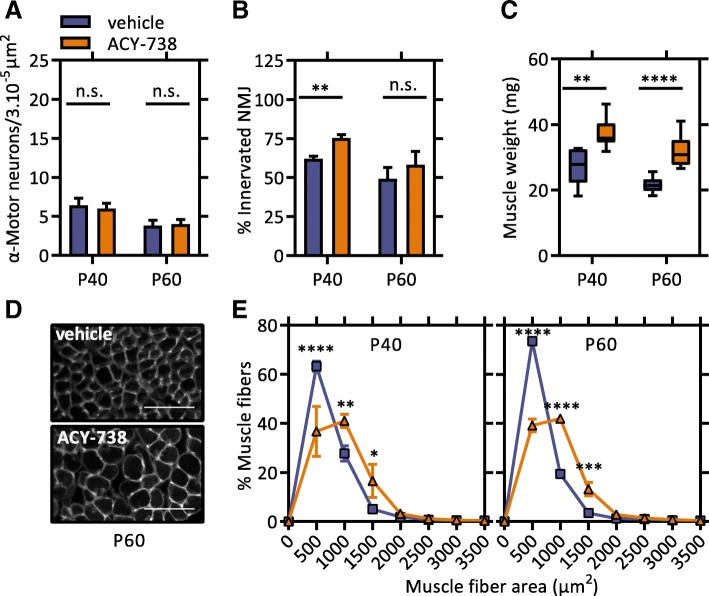
ACY-738 therapy reduces neuromuscular denervation and muscle atrophy in Tg *FUS*+/+ mice. **a** Number of α-motor neurons in the lumbar spinal cord of P40 and P60 vehicle- and ACY-738-treated Tg *FUS*+/+ mice. *n* = 4–9, Student’s *t*-test. **b** Percentage of innervated neuromuscular junctions in the gastrocnemius muscle of P40 and P60 vehicle- and ACY-738-treated Tg *FUS*+/+ mice. At least 50 neuromuscular junctions were counted per mouse. *n* = 4–6, Student’s *t*-test. **c** Weight of gastrocnemius muscle of P40 and P60 vehicle- and ACY-738-treated Tg *FUS*+/+ mice. *n* = 6–10, Student’s *t*-test. **d** Representative picture of muscle fibers of the gastrocnemius muscle P60 vehicle- and ACY-738-treated Tg *FUS*+/+ mice. Scale bar = 100 μm. (**e**) Muscle fiber area distribution of the gastrocnemius muscle of P40 and P60 vehicle- and ACY-738-treated Tg *FUS*+/+ mice. *n* = 3–4, Two-way ANOVA with Tukey’s multiple comparisons test. *P < 0.05, **P < 0.01, ***P < 0.001 ****P < 0.0001. Data are presented as means ± SEM

In order to explore the therapeutic effect of ACY-738 on the motor unit, we performed histological analyses at an early (P40) and late (P60) symptomatic age. We assessed the effect of ACY-738 on motor neuron degeneration by counting the number of α-motor neuron cell bodies in the ventral horn of the lumbar spinal cord. We observed that the ACY-738 treatment did not affect degeneration of motor neuron cell bodies at both time points (Fig. 4a). However, we found enhanced innervation of the gastrocnemius muscle in the ACY-738-treated mice compared to control Tg *FUS*+/+ mice at P40, which was less apparent at P60 (Fig. 4b). These findings suggest that ACY-738 treatment slowed down denervation.

We further evaluated the effect of ACY-738 treatment on muscle atrophy, as this reflects the functionality of the neuromuscular junctions (NMJs) [45, 55]. At both time points, the weight of the gastrocnemius muscle of ACY-738-treated Tg *FUS*+/+ mice was significantly higher than those of vehicle-treated controls (Fig. 4c). Analysis of the myofiber size distribution showed that ACY-738-treated Tg *FUS*+/+ animals had an increased number of myofibers with a large diameter compared to control animals at both time points, indicating a beneficial effect of the treatment on muscle atrophy (Fig. 4d, e). In addition to maintaining innervation, these results imply that ACY-738 treatment increased the functionality of the NMJs and/or directly supported muscle function.

Besides degeneration of the motor unit, reactive gliosis is a key pathological observation in ALS patients. We assessed the effect of ACY-738 on astrocytosis and microgliosis in the lumbar spinal cord of Tg *FUS*+/+ mice by immuno-stainings for GFAP and CD11b, respectively. These stainings confirmed gliosis in the spinal cord of Tg *FUS*+/+ mice, but could not demonstrate an obvious change of this process by ACY-738 (Additional file 5: Figure S4).

**Fig. 5:**
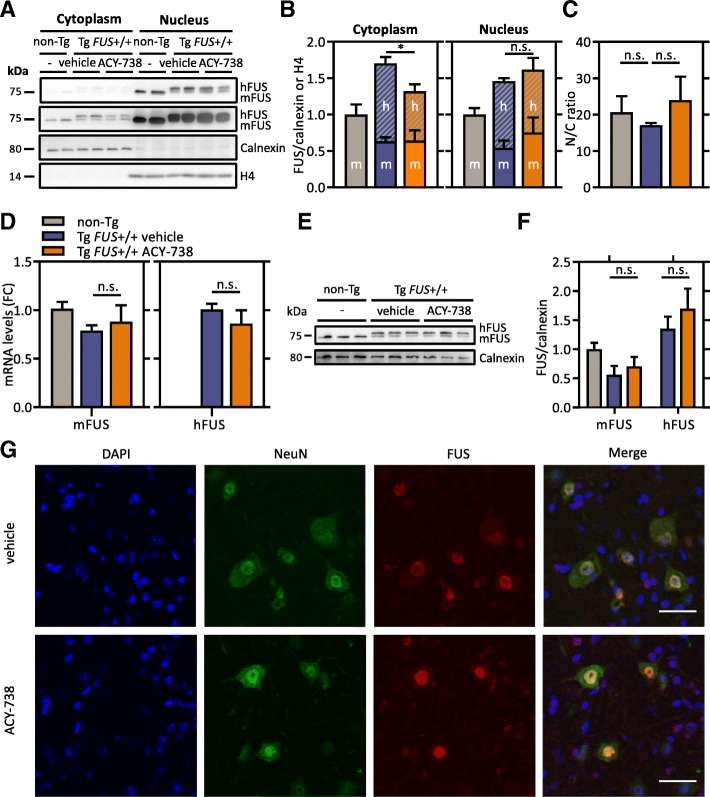
ACY-738 reduces cytoplasmic FUS levels in Tg *FUS*+/+ mice. **a** Nuclear-cytoplasmic fractionation of spinal cord tissues of P60 non-Tg controls, vehicle- and ACY-738-treated Tg *FUS*+/+ mice. The top 75 kilodalton panel is a lower contrast image of FUS, the second 75 kilodalton panel is a higher contrast image of FUS. Calnexin was used as a cytoplasmic marker and as reference for equal loading. Histone 4 was used as a nuclear marker and as reference for equal loading. **b** Quantification of the ratio of murine FUS (mFUS) and human transgene FUS (hFUS) to calnexin (cytoplasm) or histone 4 (nucleus) and normalization to non-Tg controls. *n* = 4, One-way ANOVA with Tukey’s multiple comparisons test. **c** Nuclear-to-cytoplasmic ratio calculated from absolute total FUS levels in both fractions. *n* = 4, One-way ANOVA with Tukey’s multiple comparisons test. **d** Quantitative PCR analysis of mRNA expression levels of murine and human FUS in the spinal cord of P60 non-Tg controls, vehicle- and ACY-738-treated Tg *FUS*+/+ mice, with Ap3b1 and Mon2 as reference genes and normalization to non-Tg controls. Fold change compared to non-Tg controls (FC). *n* = 6, Student’s t-test. **e** Western blot of FUS in the spinal cord of P60 Tg *FUS*+/+ mice and non-Tg controls. Calnexin levels were used as reference for equal loading. **f** Quantification of the ratio of Hdac1, Hdac2 and Hdac3 to calnexin and normalization to non-Tg controls. **g** Immunostaining of FUS and neurons (NeuN) in spinal cord of P60 vehicle- and ACY-738-treated Tg *FUS*+/+ mice. Scale bar = 35 μm. *P < 0.05. Data are presented as means ± SEM

A typical pathological hallmark that is specific for FUS-mediated ALS is cytoplasmic mislocalization of FUS in motor neurons. To investigate the effect of HDAC inhibition on this core pathological alteration, we analyzed the levels of FUS in cytoplasmic and nuclear fractions of spinal cord tissues of late symptomatic mice by immunoblotting. We could confirm increased FUS levels in the cytoplasm and nucleus as was described in the initial study. (Fig. 5a, b) [40]. Similar as in non-Tg mice, the majority of FUS still resided in the nucleus, although a slight decrease of the nuclear-to-cytoplasmic ratio was seen in Tg *FUS*+/+ mice (Fig. 5a, c). Strikingly, we observed that ACY-738-treatment significantly decreased transgene FUS levels in the cytoplasm and moderately increased its levels in the nucleus (Fig. 5a, b). This subcellular localization shift moderately increased the nuclear-to-cytoplasmic ratio (Fig. 5c). Importantly, the overall FUS levels were not affected by the treatment, which was confirmed by qPCR and Western blot analysis (Fig. 5d-f). Immunostainings for FUS in the ventral horn of the lumbar spinal cord confirmed a limited decrease in cytoplasmic FUS levels in ACY-738-treated Tg *FUS*+/+ mice compared to vehicle-treated controls (Fig. 5g).

Collectively, these results indicate that ACY-738 partially prevented cytoplasmic mislocalization of FUS in the remaining motor neurons, which could positively affect their function. This could preserve the innervation and hence improve the motor performance and survival of the Tg *FUS*+/+ mice.

### HDAC6 is not involved in the therapeutic effect of ACY-738 in Tg FUS+/+ mice

**Fig. 6:**
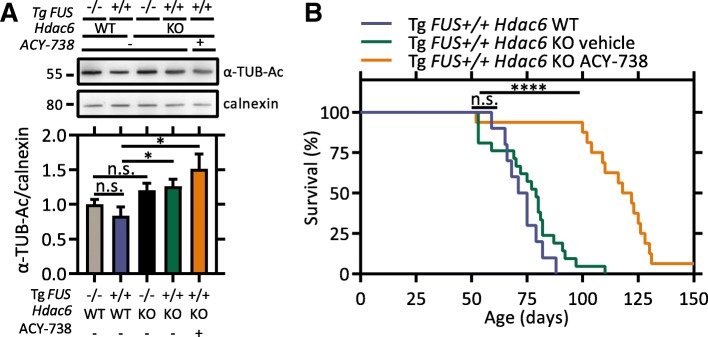
HDAC6 is not the key HDAC effector in the beneficial action of ACY-738. **a** Western blot of α-tubulin acetylation levels in the spinal cord of P60 non-Tg *Hdac6* WT, Tg *FUS*+/+ *Hdac6* WT, non-Tg *Hdac6* KO mice, and vehicle- or ACY-738-treated Tg *FUS*+/+ *Hdac6* KO mice. Calnexin levels were used as reference for equal loading. The bar graph represents the quantification of the ratio of α-tubulin acetylation to calnexin and normalization to non-Tg *Hdac6* WT controls. *n* = 3, One-way ANOVA with Tukey’s multiple comparisons test. **b** Kaplan-Meier survival analysis of Tg *FUS*+/+ *Hdac6* WT and vehicle- or ACY-738-treated Tg *FUS*+/+ *Hdac6* KO mice. Median life span of Tg *FUS*+/+ *Hdac6* WT control mice is 73 days, that of vehicle-treated Tg *FUS*+/+ *Hdac6* KO mice is 79 days, that of ACY-738-treated Tg *FUS*+/+ *Hdac6* KO mice is 120 days. *n* = 10–21, Log-rank test. *P < 0.05, ****P < 0.0001. Data are presented as means ± SEM

Besides the class I HDACs, ACY-738 is capable of inhibiting the cytoplasmic class IIb HDAC6. To rule out the possibility that HDAC6 was mediating the therapeutic effect of ACY-738, we investigated whether the suppression of HDAC6 alone phenocopied the broad-acting HDAC inhibitor ACY-738, by combining genetic and pharmacological approaches. First, we investigated the consequences of genetic removal of *Hdac6* on the disease phenotype by crossbreeding the Tg *FUS*+/+ mice with *Hdac6* knock-out (KO) mice [79]. Increased acetylation of α-tubulin can be used as a readout for HDAC6 inhibition or deletion [28]. Indeed, we confirmed that Tg *FUS*+/+; *Hdac6* KO mice showed hyperacetylated α-tubulin compared with their Tg *FUS*+/+; *Hdac6* WT littermates (Fig. 6a). However, Kaplan-Meier survival analysis could not demonstrate a longer survival after genetic *Hdac6* removal in the Tg *FUS*+/+ mice (Fig. 6b). Conversely, Tg *FUS*+/+; *Hdac6* KO mice treated with ACY-738 showed a similar extension in life span of 41 days as treated Tg *FUS*+/+ mice (Fig. 6b). The treatment regimen in KO mice did not produce further increases in α-tubulin acetylation over KO baseline levels (Fig. 6a).

These findings strongly indicate that HDAC6 is not the key HDAC effector responsible for the beneficial effect of ACY-738 in the Tg *FUS*+/+ mouse model. Collectively, our data further substantiate that the therapeutic activity of ACY-738 is mediated by the inhibition of class I HDACs.

### HDAC inhibition restores disease-associated pathways in Tg FUS+/+ mice

To elucidate the disease-causing pathways and possible treatment targets of ACY-738, we performed an integrative transcriptome and proteome analysis on spinal cord tissue of 60-day old non-Tg, vehicle- and ACY-738-treated Tg *FUS+/+* mice.

**Fig. 7:**
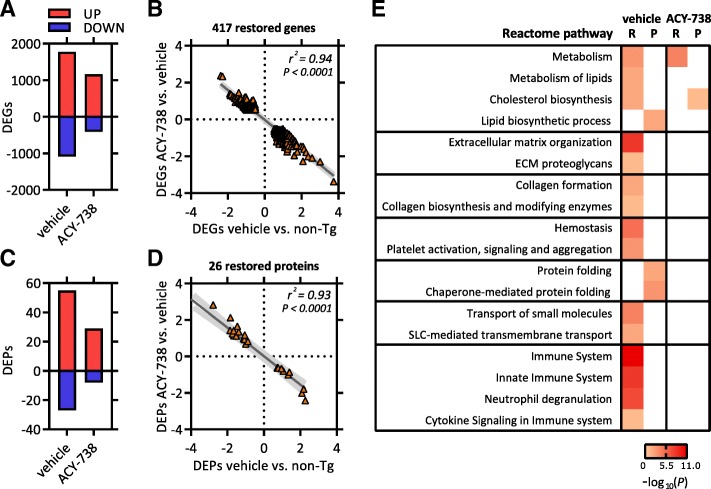
HDAC inhibition using ACY-738 restores transcriptional and proteomic alterations in spinal cord of Tg *FUS*+/+ mice. **a** Differentially expressed genes (DEGs) in vehicle- or ACY-738-treated Tg *FUS*+/+ mice compared to non-Tg controls. **b** Pearson correlation analysis on genes with completely restored expression after ACY-738 therapy. Correlation between expression values in vehicle-treated Tg *FUS*+/+ mice compared to non-Tg controls (x-axis) versus the expression values in ACY-738-treated compared to vehicle-treated Tg *FUS*+/+ mice (y-axis). **c** Differentially expressed proteins (DEPs) in vehicle- or ACY-738-treated Tg *FUS*+/+ mice compared to non-Tg controls. **d** Pearson correlation analysis on proteins with completely restored expression after ACY-738 therapy. Correlation between expression values in vehicle-treated Tg *FUS*+/+ mice compared to non-Tg controls (x-axis) versus the expression values in ACY-738-treated compared to vehicle-treated Tg *FUS*+/+ mice (y-axis). **e** Heatmap of –log_10_ *P*-values of pathways that were significantly enriched after panther gene ontology (GO) analysis on differentially expressed genes/proteins in vehicle-treated Tg *FUS*+/+ mice compared to non-Tg controls, and in ACY-738-treated Tg *FUS*+/+ mice compared to vehicle-treated Tg *FUS*+/+ mice. RNA sequencing (R), proteomics (P)

Multidimensional scaling (MDS) of the transcriptomics dataset (15,203 detected genes) indicated a high degree of gene expression similarity between biological replicates (Additional file 6: Figure S5A). Statistical analysis showed that 2860 genes were significantly differentially expressed in vehicle-treated Tg *FUS*+/+ mice compared to non-Tg controls (Fig. 7a). Comparison of ACY-738-treated Tg *FUS*+/+ mice with non-Tg revealed 1576 differentially expressed genes (DEGs), indicating that ACY-738 therapy leads to a less severe effect on overall gene expression (Fig. 7a). Heat map analysis of all differentially expressed genes revealed that ACY-738 therapy rescued a large part of the disease-induced transcription aberrations, yielding a gene expression profile more similar to non-Tg controls (Additional file 6: Figure S5B). Besides the (partial) restoration of disease-induced expression changes, a moderate number of new transcriptomic changes (111 genes) were detected after ACY-738 therapy (Additional file 6: Figure S5C).

To determine the therapeutically relevant transcriptional changes, we focused on the intersection between disease- and drug-induced expression changes, which resulted in a list of 686 genes (Additional file 6: Figure S5C). The majority showed a trend towards the wild-type level after ACY-738 treatment. A subset of 417 genes demonstrated a complete restoration after treatment (Additional file 6: Figure S5C and Fig. 7b). As the beneficial effect of ACY-738 most likely results from reinstating histone acetylation, these fully restored genes might be considered as most relevant.

The proteomics dataset, containing 3491 detected proteins, was analyzed in a similar way. MDS revealed high similarity between the 3 biological replicates (Additional file 6: Figure S5D). Statistical analysis showed that the levels of 82 proteins were significantly altered in vehicle-treated Tg *FUS*+/+ mice compared to non-Tg controls (Fig. 7c). Also here, ACY-738-treated Tg *FUS*+/+ mice presented with less (37) differentially expressed proteins (DEPs) compared to non-Tg controls (Fig. 7c). Heat map analysis of all DEPs revealed that ACY-738 therapy rescued a large part of the disease-induced protein aberrations (Additional file 6: Figure S5E). The levels of 26 proteins were fully restored to wild-type levels (Additional file 6: Figure S5F and Fig. 7d).

Next, we performed a multi-omics correlation analysis to find out whether the same trends and patterns were found with transcriptomics and proteomics analyses. We compared differential expression based on the proteomic data with the differential expression of the corresponding mRNAs using the transcriptomic data. In vehicle-treated Tg *FUS*+/+ mice, 441 of the 3491 detected proteins were significantly altered on the mRNA level compared to controls (Additional file 6: Figure S5G). Overall, 33 of the 82 DEPs could not be explained by differences on the mRNA level **(**Additional file 6: Figure S5G). A possible explanation is that these proteins are regulated at the post-transcriptional level. Pearson correlation analysis showed a modest though significant correlation of the expression values for all found protein-transcript pairs (*r*^2^ = 0.31) (Additional file 6: Figure S5H). A consistent change at the mRNA and protein level was shown for a subset of 49 genes that were significantly differentially expressed in both datasets (*r*^2^ = 0.84) (Additional file 6: Figure S5I).

In ACY-738-treated Tg *FUS*+/+ mice, 163 of the 3491 detected proteins were differentially expressed on the mRNA level compared to controls (Additional file 6: Figure S5J). Fourteen of the 37 DEPs could not be validated on the mRNA level (Additional file 6: Figure S5J). Similarly, there was a moderate but significant correlation of the expression values for all found protein-transcript pairs (*r*^2^ = 0.24) (Additional file 6: Figure S5K). Again, the subset of 23 proteins overlapping between the DEGs and DEPs showed a strong correlation with their reciprocal transcript levels (*r*^2^ = 0.75) (Additional file 6: Figure S5L).

Using the Panther Gene Ontology (GO) enrichment analysis program on both omics datasets, we found that most genes and proteins that were changed in Tg *FUS*+/+ mice were associated with inflammation, metabolism, protein folding and cellular structures (Fig. 7e) [3, 39, 66]. Analysis of the subset of genes and proteins that were fully restored after ACY-738 therapy revealed an enrichment of GO terms related to metabolism (Fig. 7e).

The observed alterations in the expression of metabolic genes in the Tg *FUS*+/+ mouse model are interesting, as increasing evidence suggests that metabolic disturbances contribute to ALS pathogenesis [71]. Targeted search of genes involved in metabolism showed decreased levels of genes associated with fatty acid and cholesterol biosynthesis and β-oxidation, and increased levels of genes associated with glycolysis, the pentose phosphate pathway (PPP) and lipid transport in Tg *FUS*+/+ mice (Additional file 7: Figure S6). ACY-738 treatment partially restored the expression levels of most of these genes, both on the mRNA and protein level (Additional file 7: Figure S6). The changes induced by the treatment include both up- and downregulated genes, suggesting an overall multifaceted restoration of these pathways.

### HDAC inhibition reinstates metabolic homeostasis in the spinal cord

**Fig. 8:**
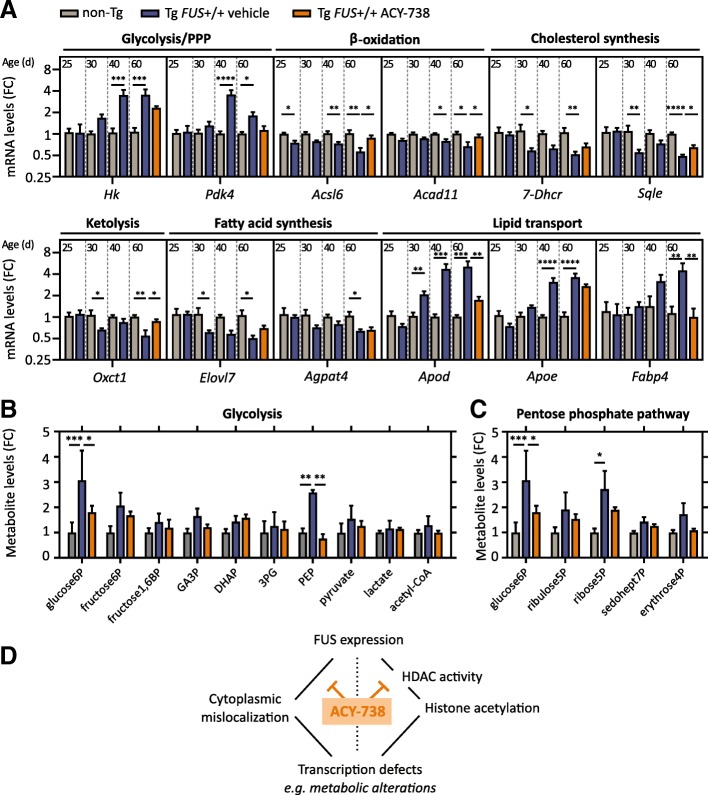
ACY-738 reinstates metabolic homeostasis in the spinal cord of Tg *FUS*+/+ mice. **a** Quantitative PCR analysis of mRNA expression levels of metabolic genes in the spinal cord of P25, P30, P40 and P60 vehicle-treated Tg *FUS*+/+ mice and non-Tg controls, and in P60 ACY-738-treated Tg *FUS*+/+ mice, with Ap3b1 and Mon2 as reference genes and normalization to non-Tg controls. *n* = 6, Student’s *t*-test with Holm-Sidak method to correct for multiple testing at P25, P30 and P40 and One-way ANOVA at P60. Pentose phosphate pathway (PPP), Hexokinase 2 (*Hk2*), pyruvate dehydrogenase kinase 4 (*Pdk4*), Acyl-CoA synthetase long-chain family member 6 (*Acsl6*), acyl-CoA dehydrogenase family member 11 (*Acad11*), 3-oxoacid CoA-transferase (*Oxct1*), 7-Dehydrocholesterol reductase (*7-Dhcr*), squalene epoxidase (*Sqle*), Elongation of very long chain fatty acids protein 7 (*Elovl7*), 1-acylglycerol-3-phosphate O-acyltransferase 4 (*Agpat4*), Apolipoprotein D (*Apod*), Apolipoprotein E (*Apoe*), Fatty acid binding protein 4 (*Fabp4*). Fold change compared to non-Tg controls (FC). **b** Metabolite levels of glycolysis intermediates in the spinal cord of P60 non-Tg controls, vehicle- and ACY-738-treated Tg *FUS*+/+ mice normalized to non-Tg controls. *n* = 3, Two-way ANOVA with Tukey’s multiple comparisons test. Glucose-6-phosphate (glucose6P), fructose-6-phosphate (fructose6P), fructose-1,6-biphosphate (fructose1,6BP), glyceraldehyde-3-phosphate (GA3P), dihydroxyacetone phosphate (DHAP), 3-phosphoglycate (3PG), phosphoenolpyruvate (PEP). **c** Metabolite levels of pentose phosphate pathway intermediates in the spinal cord of P60 non-Tg controls, vehicle- and ACY-738-treated Tg *FUS*+/+ mice normalized to non-Tg controls. *n* = 3, Two-way ANOVA with Tukey’s multiple comparisons test. Ribulose-5-phosphate (ribulose5P), ribose-5-phosphate (ribose5P), sedoheptulose-7-phosphate (sedohept7P), erythrose-4-phosphate (erythrose4P). **d** Schematic representation of our results indicating ACY-738 therapy restores histone acetylation and FUS accumulation in the cytoplasm in transgenic mice overexpressing wild-type FUS, thereby partially restoring transcriptional defects and improving the ALS phenotype. *P < 0.05, **P < 0.01, ***P < 0.001 ****P < 0.0001. Data are presented as means ± SEM

While transcriptomic and proteomic experiments took place on late-symptomatic Tg *FUS*+/+ mice, we investigated whether the observed metabolic abnormalities were critical for or rather a consequence of the disease. In order to do so, we performed qPCR experiments on spinal cord tissue of Tg *FUS*+/+ mice at pre-symptomatic age (P25), symptom onset (P30), early (P40) and late symptomatic (P60) age. Strikingly, we found that genes related to the breakdown of fatty acids were already significantly downregulated at pre-symptomatic age, whereas the downregulation of cholesterol and lipid synthesis, as well as the upregulation of lipid transport and glycolysis, were disturbed starting from symptom-onset or later (Fig. 8a). The upregulation of glycolysis and lipid transport pathways may therefore be a secondary mechanism compensating for primary defects in lipid metabolism. In P60 ACY-738-treated mice, the majority of the observed metabolic gene expression aberrations were less pronounced or even absent (Fig. 8a). These data indicate that alterations in the expression of metabolic genes are an initial and maybe critical event in the pathology of Tg *FUS*+/+ mice and suggest that ACY-738 therapy restores metabolic homeostasis.

To investigate the functional consequence of abnormalities in metabolic gene expression, we performed a metabolomic study at P60. Interestingly, several key glycolysis and PPP metabolites were significantly upregulated in the vehicle-treated Tg *FUS*+/+ mice, consistent with the observed upregulation of the enzymes that catalyze the corresponding reactions in this pathway (Fig. 8b, c and Additional file 7: Figure S6).

Collectively, these findings show that dysregulated metabolism is an early phenomenon correlating with progressive motor neuron pathology in Tg *FUS*+/+ mice and indicate that ACY-738 may act by reinstating metabolic homeostasis.

## Discussion

The pathological mechanisms underlying ALS remain incompletely understood, which could account for the numerous failed clinical trials in this patient population. We provide evidence for the contribution of aberrant histone acetylation in motor neuron pathology and discovered that HDAC inhibitors are an effective therapy in a preclinical model of ALS. Our data show that ACY-738, a potent HDAC inhibitor that crosses the BBB, restored global histone acetylation in the spinal cord, ameliorated the disease phenotype and significantly extended the lifespan of mice overexpressing wild-type *FUS*. We found that HDAC6 is not the key HDAC mediating this therapeutic effect. Our multi-omics approach revealed that dysregulated metabolism was an early phenomenon correlating with motor neuron pathology, which was largely restored by ACY-738 therapy.

Recent evidence points to a possible role of epigenetic mechanisms, including microRNAs, DNA methylation and histone modifications, in ALS pathology [7, 44]. Amongst these, we concentrated on the possible contribution of histone acetylation, a post-translational modification that regulates transcription, in a rodent model of ALS. Aberrant activation of HDACs, resulting in histone hypoacetylation, has been associated with several neurodegenerative disorders and with neuronal toxicity [7, 31, 58]. Pioneering work by Rouaux and collaborators reported evidence for a role of HDACs in ALS, demonstrating histone hypoacetylation in the SOD1 mouse model starting at the age of onset [53]. In postmortem brain and spinal cord specimens of ALS patients, analysis of HDAC expression levels also revealed altered levels of HDAC2 and HDAC11 [29]. While the various HDACs were classified and categorized, the role of particular HDACs in ALS pathogenesis has not been fully addressed yet. In more recent studies, it was shown that muscle HDAC4 plays a crucial role in muscle reinnervation in SOD1 mice and in patients with rapidly progressive ALS [11, 48, 49, 74].

One important question is the molecular mechanism underlying the decrease in histone acetylation observed in Tg *FUS*+/+ mice. ALS-causative genes have been associated with various epigenetic modifiers and epigenetic tags [7, 12]. For example, the DNA/RNA binding protein FUS directly interacts with CBP and p300, two histone acetyltransferases, as well as with HDAC1 [72, 73]. Therefore, the observed hyperactivity of nuclear HDACs and associated histone hypoacetylation in the Tg *FUS*+/+ mice could arise from such an interaction. Further studies are required to confirm this.

The role of HDACs in neurodegenerative diseases has largely been deduced from effects observed after inhibition of their enzymatic activity. In the context of ALS, several studies have shown a modest protective effect of three pan-HDAC inhibitors, trichostatin A (TSA), valproic acid (VPA) and phenyl butyrate (PB) in the SOD1 model [15, 46, 56, 76]. TSA is a very potent HDAC inhibitor, yet it can be used only in laboratory experiments due to its genotoxic effects [43]. VPA is used as an antiepileptic drug, but has a low HDAC inhibitory potency, poor BBB permeability and cumbersome side effects. PB is currently used to treat hyperammonemia due to urea cycle disorders and is also known to have a low inhibitory potency. It may therefore not be surprising that phase II clinical trials in ALS patients showed negative results [13, 47]. In our study, we used ACY-738 to explore the therapeutic potential of HDAC inhibition in a preclinical *FUS* model of ALS. ACY-738 is a highly BBB permeable, potent HDAC inhibitor that primarily targets class I HDACs and HDAC6 [8, 30, 41]. Our data show that the inhibition of the class I HDACs, which corrects global histone acetylation and partially restores transcription, is responsible for the positive effect in the Tg *FUS*+/+ model. ACY-738 is an attractive drug candidate, as we could show robust class I HDAC inhibition in vivo with no obvious adverse effects on the treated animals.

The finding that genetic removal of *Hdac6* did not have a beneficial effect in the Tg *FUS*+/+ mice is in contrast with our results observed in the SOD1 model, in which HDAC6 removal modestly extended their survival [65]. This could be due to differences in the pathogenic mechanisms underlying both genetic forms of ALS. Another indication of this diversity is our previous observation that pharmacological inhibition as well as genetic silencing of HDAC6 in a different FUS-related model had positive effects [27]. Motor neurons differentiated from induced pluripotent stem cells (iPSCs) from *FUS*-ALS patients developed axonal transport defects over time and inhibition of HDAC6 using tubastatin A or ACY-738 completely rescued these defects [27]. In our current study, we excluded that HDAC6 inhibition by ACY-738 was responsible for the positive effect on the survival of the Tg *FUS*+/+ mice. One should keep in mind that these different effects of ACY-738 were obtained in two completely different models that both mimic relevant aspects of the ALS disease process. Whatever the exact contribution in ALS patients is of axonal transport defects due to decreased acetylation of α-tubulin or of aberrant transcription caused by hypoacetylation of histones, ACY-738 has the major advantage that it corrects both processes.

Furthermore, we noticed that ACY-738 therapy can significantly decrease cytoplasmic human FUS levels in the remaining motor neurons. It is possible that ACY-738 acts on this process by slowing down several key aspects of disease progression due to its effects on transcription. However, the partial restoration of FUS localization might also be independent from changes in histone acetylation or transcription. The synergistic correction of each of these different processes could contribute to the therapeutic activity of ACY-738.

Using an integrative transcriptomic, proteomic and metabolomic approach, we ultimately discovered that dysregulation of lipid metabolism was an early phenomenon correlating with progressive motor neuron pathology. These results are in line with a recent study that showed drastic morphological alterations in mitochondria at the synaptic terminals of pre-symptomatic Tg *FUS*+/+ mice [62]. Furthermore, expression changes of genes related to (lipid) metabolism were noted in another wild-type FUS overexpression mouse model, as well as in a mutant FUS overexpression and FUS-ΔNLS-knock-in mouse model, suggesting a specific pathogenic mechanism contributing to FUS-mediated ALS [17, 50, 61]. It is possible that these other FUS models also present with histone acetylation changes that dysregulate the expression of metabolic genes. Strikingly, our multi-omics analysis revealed a substantial rescue of the observed metabolic disturbances by ACY-738 treatment. Although neurons and glial cells express many molecular components of lipid metabolic pathways, the importance of local regulation of lipid metabolism in the CNS has remained neglected for a long time. Only recently, several studies highlight central lipid metabolism homeostasis as a critical factor for neuronal function and survival [33, 35, 36]. Cholesterol is locally synthesized in the adult CNS, with little or no import from the periphery [77]. It is synthesized mostly by astrocytes, after which it is transported by apolipoprotein E (APOE) and taken up by neurons via the low-density lipoprotein receptor. Fatty acids (FA) on the other hand are mainly transported into the CNS from the systemic circulation, although some FAs can be synthesized de novo by neurons and astrocytes [10, 23, 35]*.* Disturbances in either the synthesis, transport, or turnover of lipids were shown to trigger synapse loss and neurodegeneration [10, 23, 25, 35, 36]. The observed disturbances in lipid metabolic pathways in the Tg *FUS*+/+ mice could affect membrane biogenesis-dependent processes such as synaptogenesis, synapse maintenance, neurotransmitter release and mitochondrial function [26, 33, 36]. As these processes are all determinants of proper neuromuscular functionality, an early disturbance in central lipid homeostasis could contribute to ALS pathogenesis [4, 60]. Accordingly, the rescue of metabolic homeostasis by ACY-738 therapy could account for the preservation of neuromuscular junctions, as appreciated by an increased number of innervated junctions and increased compound muscle action potentials.

It will be important to validate our findings on disturbed histone acetylation and metabolism in other ALS models. In fact, none of the ALS rodent models, including the Tg *FUS*+/+ mouse model used in our study, fully recapitulates human disease. Therefore, it is reasonable that only the combined knowledge obtained from each of these models will enable us to understand the deleterious processes underlying the selective motor neuron death in ALS.

Overall, our hypothesis illustrated in Fig. 8d is that ALS-associated proteins such as FUS can influence the epigenetic code. These epigenetic alterations, in combination with cytoplasmic accumulation of ALS-associated proteins, will have tremendous implications on the global transcription profile. This will affect different processes, such as metabolic pathways, that are essential for motor neurons, ultimately leading to degeneration. We propose that inhibition of class I HDACs by ACY-738 can partially overcome these transcriptional defects by restoring histone acetylation and FUS mislocalization, thereby ultimately improving the ALS phenotype. It will be important to define which HDAC family member is most crucial for the observed effect, so that safe, selective and potent HDAC inhibitors can be developed.

## Conclusions

In summary, our findings confirm that epigenetic mechanisms contribute to ALS pathology in a FUS animal model that recapitulates many aspects of the disease. Moreover, our study provides evidence that class I HDACs are promising pharmacological targets for ALS. ACY-738 showed the most significant beneficial effects of all HDAC inhibitors tested in ALS models so far, probably due to its superior pharmacokinetic features. Therefore, this drug should be further evaluated for its clinical efficacy in the context of ALS.

## Additional files

Additional file 1: **Table S1.** Primer sequences for the determination of expression of metabolic genes. Primers for quantitative PCR analysis of expression levels of metabolic genes. (DOCX 14 kb)

Additional file 2: **Figure S1.** In vitro and in vivo pharmacokinetic properties of ACY-738. (**A**) Western blots showing the effects of different concentrations of ACY-738 on acetylation of α-tubulin and histone 3 in N2a cells. (**B**) Dose-response curves of the effect of ACY-738 based on the quantification of Western blots. The ratio of acetylated α-tubulin is normalized to calnexin (top) and acetylation of histone 3 is normalized to histone 4 (bottom). Values were normalized to vehicle (*n* = 2). (**C**) Dose-response curve showing the effect of ACY-738 on acetylation of histone 3 in nuclear fractions of spinal cord lysates of non-Tg mice using a colorimetric HDAC activity kit. The orange band depicts the concentration range of the compound as measured in tissues of treated Tg *FUS*+/+ mice. (**D**) Concentrations of ACY-738 in the plasma and brain of Tg *FUS*+/+ mice measured by mass spectrometry. Half maximal inhibitory concentration (IC_50_) (*n* = 4). (PDF 73 kb)

Additional file 3: **Figure S2.** ACY-738 therapy ameliorates ALS disease phenotype both in male and female Tg *FUS*+/+ mice. (**A**) Kaplan-Meier survival analysis of vehicle- and ACY-738-treated Tg *FUS*+/+ mice split up by gender. (**B**) Overview of median life span of vehicle-treated Tg *FUS*+/+ mice and ACY-738-treated Tg *FUS*+/+ mice by gender. *n* = 23-25, of which 12 males and 11 females in the vehicle-treated group and 10 males and 15 females in the ACY-738-treated group, Log-rank test. (**C**) Longitudinal follow-up of weight and (**D**) forelimb grip strength, test of vehicle- and ACY-738-treated Tg *FUS*+/+ mice. *n* = 10–14, mice were followed up twice a week, Student’s *t*-test with Holm Sidak method to correct for multiple testing. **P < 0.01, ***P < 0.001, ****P < 0.0001. Data are presented as means ± SEM. (PDF 47 kb)

Additional file 4: **Figure S3.** Therapy with an ACY-1090, an inactive form of ACY-738, does not have any effect on ALS disease progression in Tg *FUS*+/+ mice. (**A**) Chemical structure of ACY-738 (left) and ACY-1090 (right). The active zinc-binding group of ACY-738 and the inactive form of ACY-1090 are marked in red boxes. (**B**) Kaplan-Meier survival analysis of vehicle- and ACY-1090-treated Tg *FUS*+/+ mice. (**C**) Longitudinal follow-up of weight, (**D**) forelimb grip strength, and (**E**) compound muscle action potential (CMAP) amplitudes of vehicle- and ACY-1090-treated Tg *FUS*+/+ mice. *n* = 8–10, mice were followed up twice a week, Student’s *t*-test with Holm-Sidak method to correct for multiple testing. Data are presented as means ± SEM. (PDF 50 kb)

Additional file 5: **Figure S4.** ACY-738 does not affect gliosis in the spinal cord of Tg *FUS*+/+ mice. Immunostaining for astrogliosis and microgliosis in the ventral horn of the lumbar spinal cord of P60 non-Tg controls, vehicle- and ACY-738-treated Tg *FUS*+/+ mice. GFAP was used as a marker for astrogliosis, CD11b as a marker for microgliosis. Scale bar = 100 μm. (PDF 4055 kb)

Additional file 6: **Figure S5.** Transcriptomics and proteomics analyses show a high degree of similarity. (**A**) Multidimensional scaling (MDS) of transcriptomics data. (**B**) Heat map by hierarchical clustering of all differentially expressed genes. (**C**) Venn diagram representing the overlap of differentially expressed genes between vehicle-treated Tg *FUS*+/+ mice and non-Tg controls (blue), between ACY-738-treated Tg *FUS*+/+ mice and non-Tg controls (light orange) and between vehicle- and ACY-738-treated Tg *FUS*+/+ mice (dark orange). (**D**) MDS of proteomics data. (**E**) Heat map by hierarchical clustering of all differentially expressed proteins. (**F**) Venn diagram representing the overlap of differentially expressed proteins between vehicle-treated Tg *FUS*+/+ mice and non-Tg controls (blue), between ACY-738-treated Tg *FUS*+/+ mice and non-Tg controls (light orange) and between vehicle- and ACY-738-treated Tg *FUS*+/+ mice (dark orange). (**G**) Venn diagram comparing differential expression in vehicle-treated Tg *FUS*+/+ mice compared to non-Tg controls on the mRNA- (blue grid) and protein-level (blue lines) of all identified mRNA-protein pairs. (**H**) Correlation plot of all identified mRNA-protein pairs, comparing the expression values on the mRNA and protein level in vehicle-treated Tg *FUS*+/+ mice compared to non-Tg controls. (**I**) Correlation plot of genes that were differentially expressed in both data sets, comparing the expression values on the mRNA and protein level in vehicle-treated Tg *FUS*+/+ mice compared to non-Tg controls. (**J**) Venn diagram comparing differential expression in ACY-738-treated Tg *FUS*+/+ mice compared to non-Tg controls on the mRNA- (orange grid) and protein-level (orange lines) of all identified mRNA-protein pairs. (**K**) Correlation plot of all identified mRNA-protein pairs, comparing the expression values on the mRNA and protein level in ACY-738-treated Tg *FUS*+/+ mice compared to non-Tg controls. (**L**) Correlation plot of genes that were differentially expressed in both datasets, comparing the expression values on the mRNA and protein level in ACY-738-treated Tg *FUS*+/+ mice compared to non-Tg controls. (PDF 225 kb)

Additional file 7: **Figure S6.** The expression of metabolic genes is partially restored by ACY-738 therapy. Heatmap of expression changes of a subset of metabolic genes at the mRNA and protein level based on transcriptomics and proteomics experiments. Hexokinase 2 (*Hk2*), pyruvate dehydrogenase kinase 4 (*Pdk4*), Acyl-CoA synthetase long-chain family member 6 (*Acsl6*), acyl-CoA dehydrogenase family member 11 (*Acad11*), 3-oxoacid CoA-transferase (*Oxct1*), 7-Dehydrocholesterol reductase (*7-Dhcr*), squalene epoxidase (*Sqle*), Elongation of very long chain fatty acids protein 7 (*Elovl7*), 1-acylglycerol-3-phosphate O-acyltransferase 4 (*Agpat4*), Apolipoprotein D (*Apod*), Apolipoprotein E (*Apoe*), Fatty acid binding protein 4 (*Fabp4*). (PDF 62 kb)

## Data Availability

The datasets generated during and/or analysed during the current study are available from the corresponding author on reasonable request.

## Abbreviations

ALS: Amyotrophic lateral sclerosis
APOE: Apolipoprotein E
BBB: Blood-brain-barrier
C9orf72: Chromosome 9 open reading frame 72
CMAP: Compound muscle action potential
CNS: Central nervous system
DEG: Differentially expressed gene
DEP: Differentially expressed protein
FA: Fatty acid
FTD: Frontotemporal dementia
FUS: Fused in sarcoma
GO: Gene ontology
HAT: Histone acetyltransferase
HDAC: Histone deacetylase
KO: Knock-out
MDS: Multidimensional scaling
NMJ: Neuromuscular junction
PB: Phenyl butyrate
PFA: Paraformaldehyde
PPP: Pentose phosphate pathway
RT: Room temperature
SOD1: Superoxide dismutase 1
TDP-43: TAR DNA-binding protein 43
TG: Transgenic
TSA: Trichostatin A
VPA: Valproic acid
