# Supplementary material for: Restoration of histone acetylation ameliorates disease and metabolic abnormalities in a FUS mouse model
Source: Acta Neuropathol Commun. 2019 Jul 5;7:107. doi: 10.1186/s40478-019-0750-2 (PMC6612190; doi:10.1186/s40478-019-0750-2)
Supplement: Supplementary file 1 — Table S1. Primer sequences for the determination of expression of metabolic genes. Primers for quantitative PCR analysis of expression levels of metabolic genes. (DOCX 14 kb) [file 40478_2019_750_MOESM1_ESM.docx]

| **Gene ID** | **Full name** | **Forward primer sequence** | **Reverse primer sequence** | **Source** |
| --- | --- | --- | --- | --- |
| *Hk2* | *Hexokinase 2* | GTGTGCTCCGAGTAAGGGTG | CAGGCATTCGGCAATGTGG | primerbank: primer pair 2 (163965442c2) |
| *Pdk4* | *Pyruvate dehydrogenase kinase 4* | AGGGAGGTCGAGCTGTTCTC | GGAGTGTTCACTAAGCGGTCA | primerbank: primer pair 4 (7305375a1) |
| *Acsl6* | *Acyl-CoA synthetase long chain family member 6* | AAGTGACAGAGAGTCAGTGGG | TAGGGCGGAGAGCCTTCAT | primerbank: primer pair 1 (21450203a1) |
| *Acad11* | *Acyl-CoA dehydrogenase* | AGATGCTTCAGTTATCGGAACG | ATGTAGCCATGCCAGGGTTTC | primerbank: primer pair 4 (28280023a1) |
| *Oxct1* | *3-Oxoacid CoA-transferase 1* | CATAAGGGGTGTGTCTGCTACT | GCAAGGTTGCACCATTAGGAAT | primerbank: primer pair 4 (18266680a1) |
| *7-Dhcr* | *7-Dehydrocholesterol reductase* | AGGCTGGATCTCAAGGACAAT | GCCAGACTAGCATGGCCTG | primerbank: primer pair 4 (6681179a1) |
| *Sqle* | *Squalene epoxidase* | AGTTCGCTGCCTTCTCGGATA | GCTCCTGTTAATGTCGTTTCTGA | primerbank: primer pair 1 (118130480c1) |
| *Elovl7* | *Long chain fatty acyl elongase* | CATCGAGGACTGTGCGTTTTT | CCAGGATGATGGTTTGTGGCA | primerbank: primer pair 4 (31542038a1) |
| *Agpat4* | *1-Acylglycerol-3-phosphate O-acyltransferase* | CCAGTTTCTATGTCACCTGGTC | GCAGAGTCTGGCATTGATCTTG | primerbank: primer pair 4 (27229064a1) |
| *Apod* | *Apolipoprotein D* | TCACCACAGCCAAAGGACAAA | CGTTCTCCATCAGCGAGTAGT | primerbank: primer pair 2 (6680706a1) |
| *Apoe* | *Apolipoprotein E* | CTCCCAAGTCACACAAGAACTG | CCAGCTCCTTTTTGTAAGCCTTT | primerbank: primer pair 1 (163644328c1) |
| *Fabp4* | *Fatty acid binding protein 4* | GAAGCTTGTCTCCAGTGA | GCCCAGTTTGAAGGAAAT | own design |

**Additional file 1: Table S1. Primer sequences for the determination of expression of metabolic genes.**

Primers for quantitative PCR analysis of expression levels of metabolic genes.
