# Supplementary figures and images for: Restoration of histone acetylation ameliorates disease and metabolic abnormalities in a FUS mouse model
Source: Acta Neuropathol Commun. 2019 Jul 5;7:107. doi: 10.1186/s40478-019-0750-2 (PMC6612190; doi:10.1186/s40478-019-0750-2)

**A**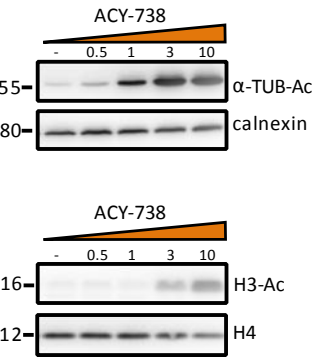**B**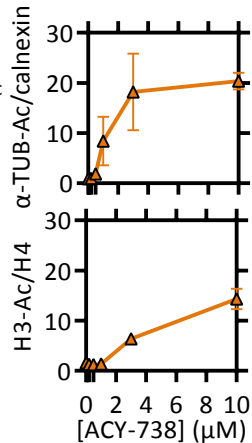**C**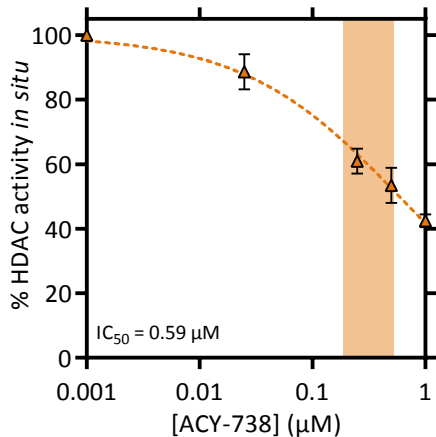**D**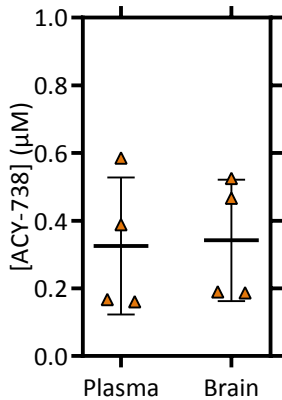

Supplement: Supplementary file 2 — Figure S1. In vitro and in vivo pharmacokinetic properties of ACY-738. (A) Western blots showing the effects of different concentrations of ACY-738 on acetylation of α-tubulin and histone 3 in N2a cells. (B) Dose-response curves of the effect of ACY-738 based on the quantification of Western blots. The ratio of acetylated α-tubulin is normalized to calnexin (top) and acetylation of histone 3 is normalized to histone 4 (bottom). Values were normalized to vehicle (n = 2). (C) Dose-response curve showing the effect of ACY-738 on acetylation of histone 3 in nuclear fractions of spinal cord lysates of non-Tg mice using a colorimetric HDAC activity kit. The orange band depicts the concentration range of the compound as measured in tissues of treated Tg FUS+/+ mice. (D) Concentrations of ACY-738 in the plasma and brain of Tg FUS+/+ mice measured by mass spectrometry. Half maximal inhibitory concentration (IC50) (n = 4). (PDF 73 kb) [file 40478_2019_750_MOESM2_ESM.pdf]

**A**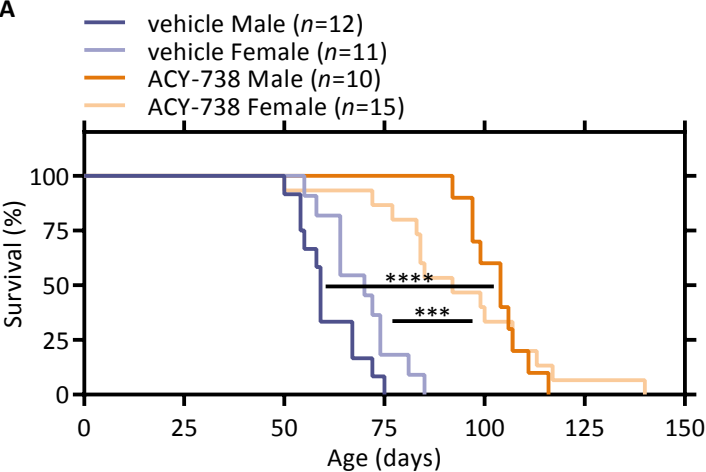**B**

|                | Male | Female |
|----------------|------|--------|
| <b>vehicle</b> | 59   | 70     |
| <b>ACY-738</b> | 104  | 92     |

**C**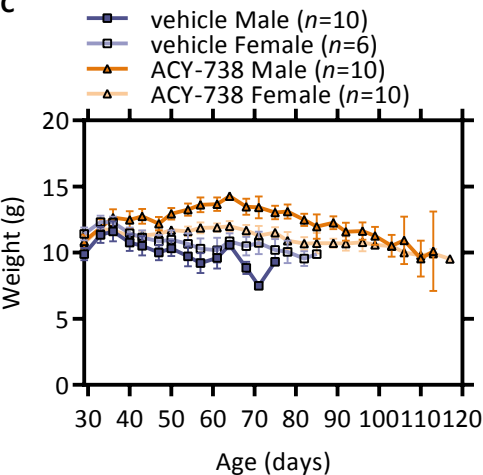**D**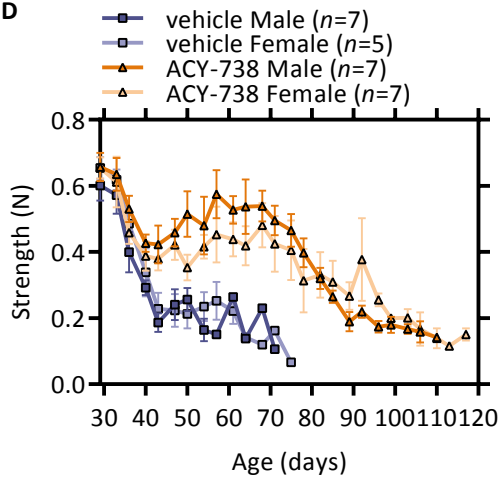

Supplement: Supplementary file 3 — Figure S2. ACY-738 therapy ameliorates ALS disease phenotype both in male and female Tg FUS+/+ mice. (A) Kaplan-Meier survival analysis of vehicle- and ACY-738-treated Tg FUS+/+ mice split up by gender. (B) Overview of median life span of vehicle-treated Tg FUS+/+ mice and ACY-738-treated Tg FUS+/+ mice by gender. n = 23-25, of which 12 males and 11 females in the vehicle-treated group and 10 males and 15 females in the ACY-738-treated group, Log-rank test. (C) Longitudinal follow-up of weight and (D) forelimb grip strength, test of vehicle- and ACY-738-treated Tg FUS+/+ mice. n = 10–14, mice were followed up twice a week, Student’s t-test with Holm Sidak method to correct for multiple testing. **P < 0.01, ***P < 0.001, ****P < 0.0001. Data are presented as means ± SEM. (PDF 47 kb) [file 40478_2019_750_MOESM3_ESM.pdf]

**A****ACY-738**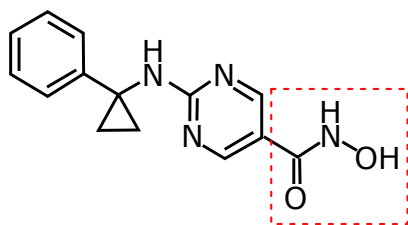**ACY-1090**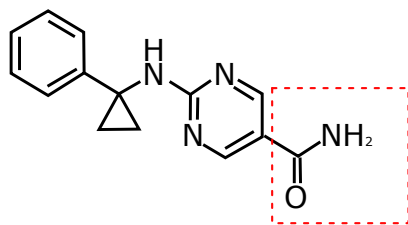**B**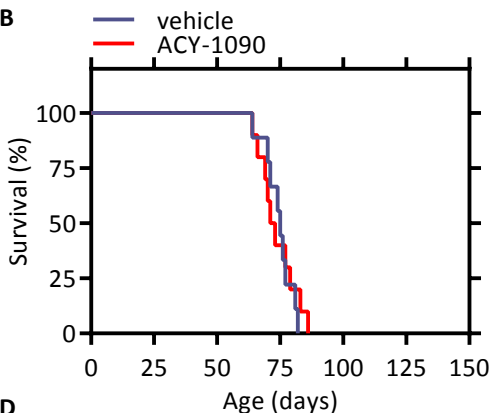**C**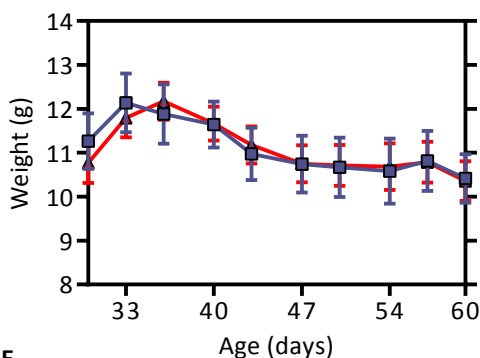**D**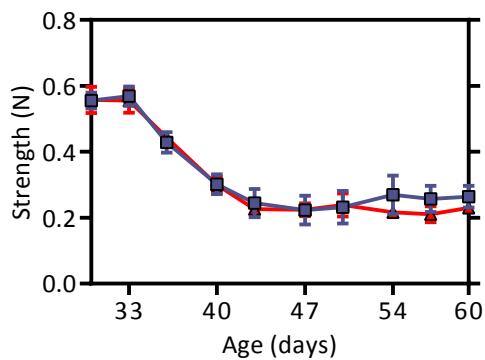**E**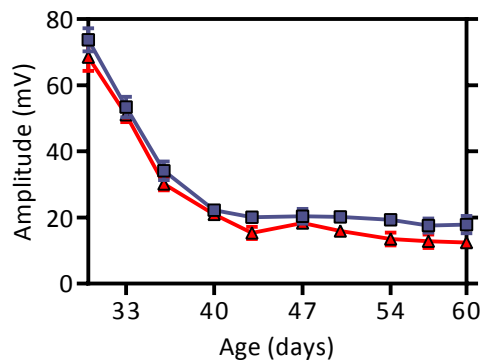

Supplement: Supplementary file 4 — Figure S3. Therapy with an ACY-1090, an inactive form of ACY-738, does not have any effect on ALS disease progression in Tg FUS+/+ mice. (A) Chemical structure of ACY-738 (left) and ACY-1090 (right). The active zinc-binding group of ACY-738 and the inactive form of ACY-1090 are marked in red boxes. (B) Kaplan-Meier survival analysis of vehicle- and ACY-1090-treated Tg FUS+/+ mice. (C) Longitudinal follow-up of weight, (D) forelimb grip strength, and (E) compound muscle action potential (CMAP) amplitudes of vehicle- and ACY-1090-treated Tg FUS+/+ mice. n = 8–10, mice were followed up twice a week, Student’s t-test with Holm-Sidak method to correct for multiple testing. Data are presented as means ± SEM. (PDF 50 kb) [file 40478_2019_750_MOESM4_ESM.pdf]

non-Tg

Tg *FUS*<sup>+/+</sup> vehicle

Tg *FUS*<sup>+/+</sup> ACY-738

GFAP

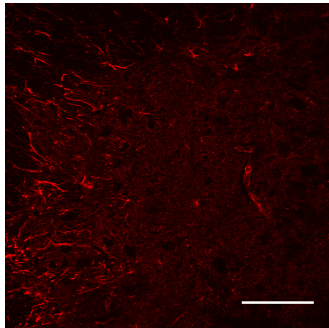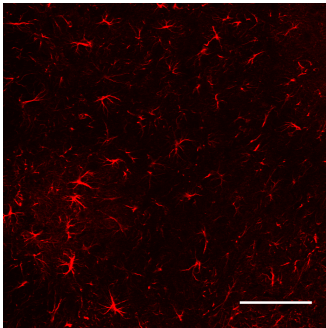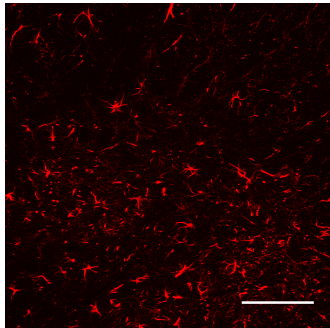

cd11b

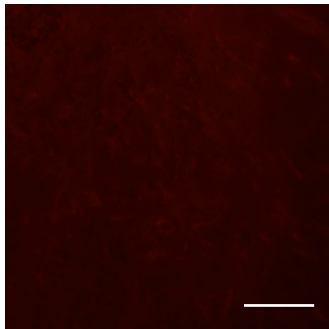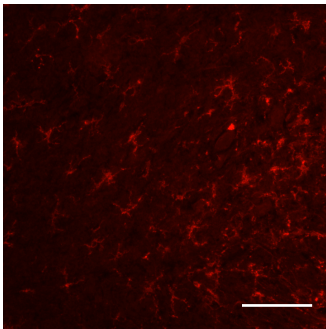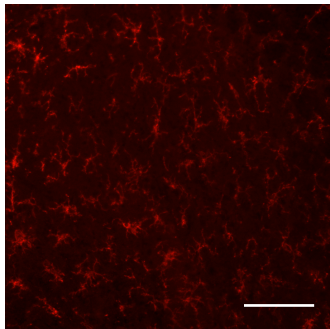

Supplement: Supplementary file 5 — Figure S4. ACY-738 does not affect gliosis in the spinal cord of Tg FUS+/+ mice. Immunostaining for astrogliosis and microgliosis in the ventral horn of the lumbar spinal cord of P60 non-Tg controls, vehicle- and ACY-738-treated Tg FUS+/+ mice. GFAP was used as a marker for astrogliosis, CD11b as a marker for microgliosis. Scale bar = 100 μm. (PDF 4055 kb) [file 40478_2019_750_MOESM5_ESM.pdf]

## TRANSCRIPTOMICS

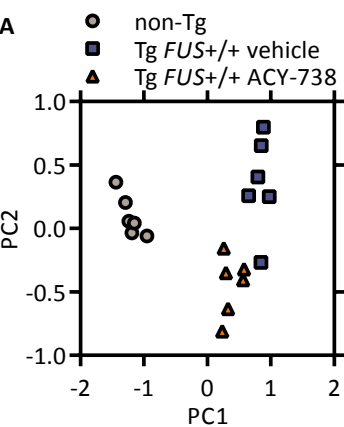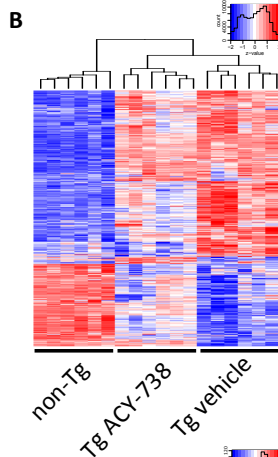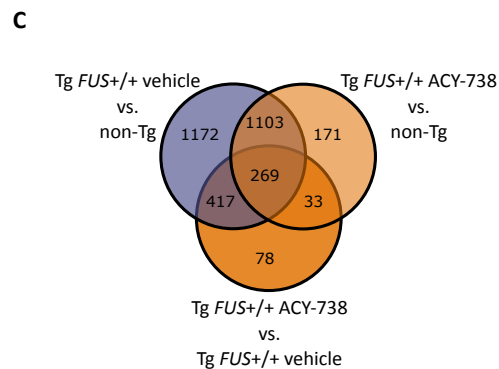

## PROTEOMICS

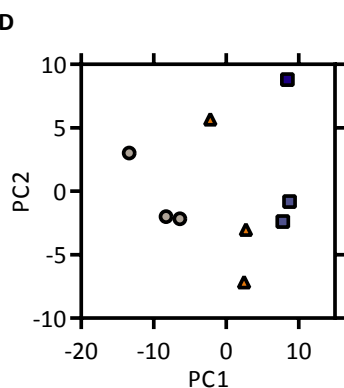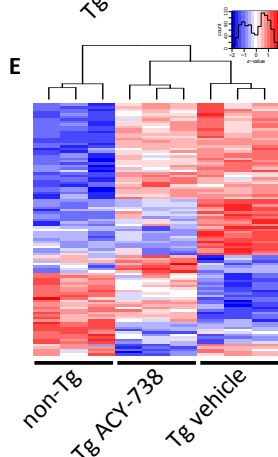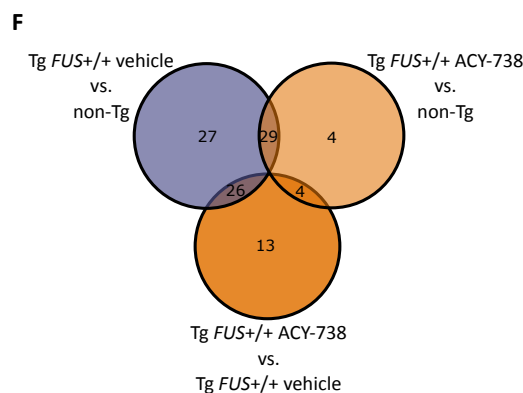

## TRANSCRIPTOMICS vs. PROTEOMICS

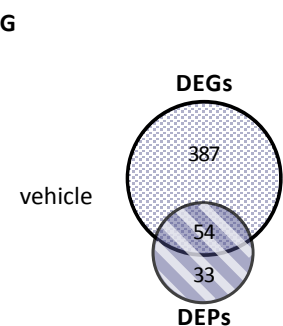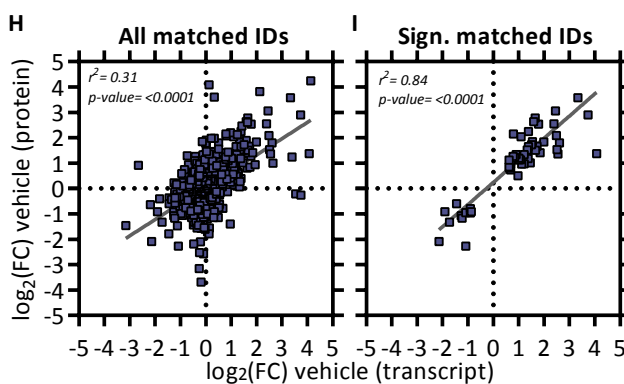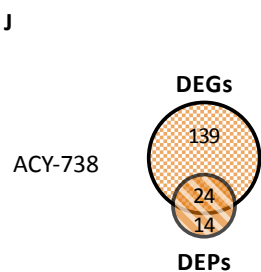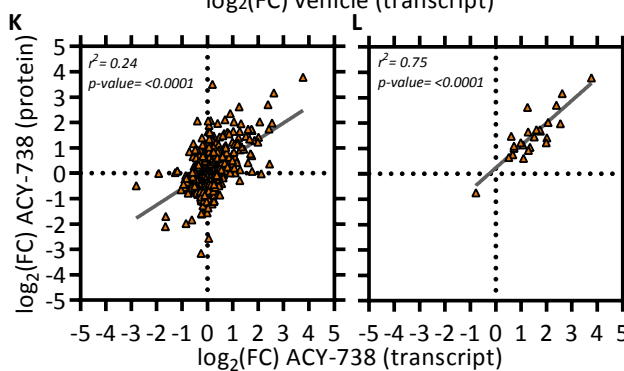

Supplement: Supplementary file 6 — Figure S5. Transcriptomics and proteomics analyses show a high degree of similarity. (A) Multidimensional scaling (MDS) of transcriptomics data. (B) Heat map by hierarchical clustering of all differentially expressed genes. (C) Venn diagram representing the overlap of differentially expressed genes between vehicle-treated Tg FUS+/+ mice and non-Tg controls (blue), between ACY-738-treated Tg FUS+/+ mice and non-Tg controls (light orange) and between vehicle- and ACY-738-treated Tg FUS+/+ mice (dark orange). (D) MDS of proteomics data. (E) Heat map by hierarchical clustering of all differentially expressed proteins. (F) Venn diagram representing the overlap of differentially expressed proteins between vehicle-treated Tg FUS+/+ mice and non-Tg controls (blue), between ACY-738-treated Tg FUS+/+ mice and non-Tg controls (light orange) and between vehicle- and ACY-738-treated Tg FUS+/+ mice (dark orange). (G) Venn diagram comparing differential expression in vehicle-treated Tg FUS+/+ mice compared to non-Tg controls on the mRNA- (blue grid) and protein-level (blue lines) of all identified mRNA-protein pairs. (H) Correlation plot of all identified mRNA-protein pairs, comparing the expression values on the mRNA and protein level in vehicle-treated Tg FUS+/+ mice compared to non-Tg controls. (I) Correlation plot of genes that were differentially expressed in both data sets, comparing the expression values on the mRNA and protein level in vehicle-treated Tg FUS+/+ mice compared to non-Tg controls. (J) Venn diagram comparing differential expression in ACY-738-treated Tg FUS+/+ mice compared to non-Tg controls on the mRNA- (orange grid) and protein-level (orange lines) of all identified mRNA-protein pairs. (K) Correlation plot of all identified mRNA-protein pairs, comparing the expression values on the mRNA and protein level in ACY-738-treated Tg FUS+/+ mice compared to non-Tg controls. (L) Correlation plot of genes that were differentially expressed in both datasets, comparing [file 40478_2019_750_MOESM6_ESM.pdf]

# TRANSCRIPTOMICS

# PROTEOMICS

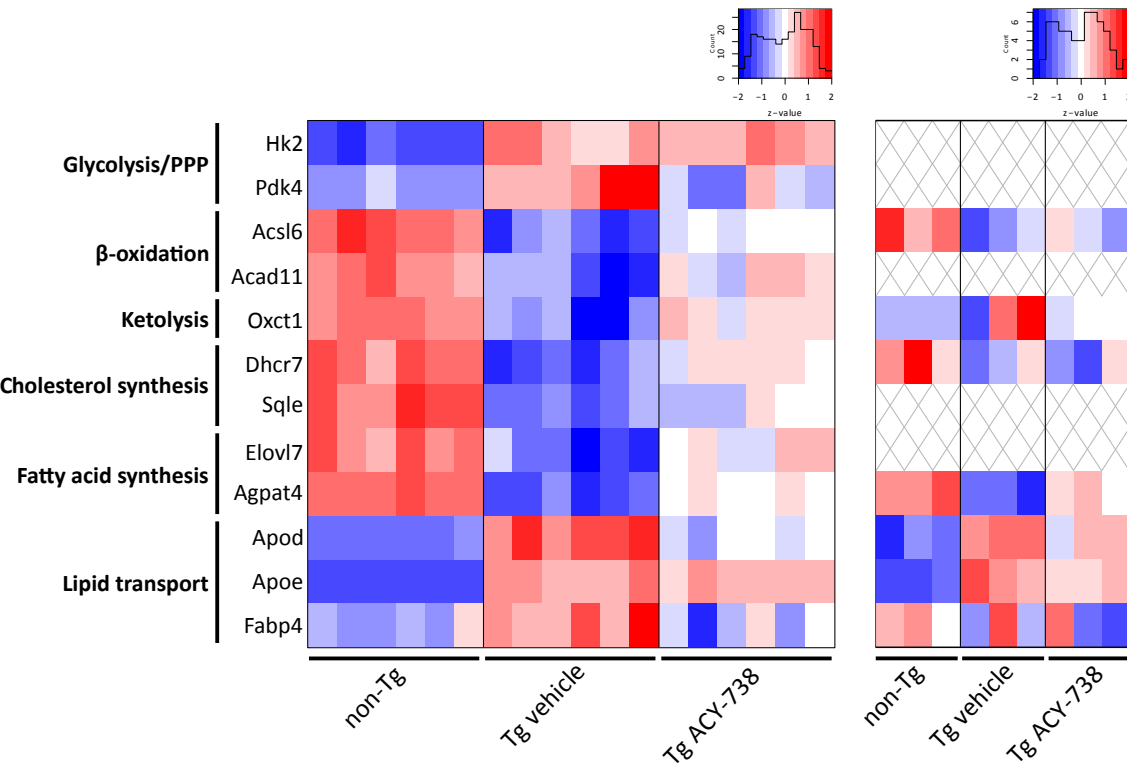

Supplement: Supplementary file 7 — Figure S6. The expression of metabolic genes is partially restored by ACY-738 therapy. Heatmap of expression changes of a subset of metabolic genes at the mRNA and protein level based on transcriptomics and proteomics experiments. Hexokinase 2 (Hk2), pyruvate dehydrogenase kinase 4 (Pdk4), Acyl-CoA synthetase long-chain family member 6 (Acsl6), acyl-CoA dehydrogenase family member 11 (Acad11), 3-oxoacid CoA-transferase (Oxct1), 7-Dehydrocholesterol reductase (7-Dhcr), squalene epoxidase (Sqle), Elongation of very long chain fatty acids protein 7 (Elovl7), 1-acylglycerol-3-phosphate O-acyltransferase 4 (Agpat4), Apolipoprotein D (Apod), Apolipoprotein E (Apoe), Fatty acid binding protein 4 (Fabp4). (PDF 62 kb) [file 40478_2019_750_MOESM7_ESM.pdf]
